# The genome of *Citrus australasica* reveals disease resistance and other species specific genes

**DOI:** 10.1186/s12870-024-04988-8

**Published:** 2024-04-10

**Authors:** Upuli Nakandala, Agnelo Furtado, Ardashir Kharabian Masouleh, Malcolm W. Smith, Darren C. Williams, Robert J. Henry

**Affiliations:** 1 Queensland Alliance for Agriculture and Food Innovation, University of Queensland, Brisbane, 4072 Australia; 2 ARC Centre of Excellence for Plant Success in Nature and Agriculture, University of Queensland, Brisbane, 4072 Australia; 3 Department of Agriculture and Fisheries, Bundaberg Research Station, Bundaberg, QLD 4670 Australia; 4 Herbalistics Pty Ltd, Bli Bli, Queensland, 4560 Australia

**Keywords:** Chromosome scale genome, Haplotype-resolved, *C. australasica* specific genes, Disease resistance, Colour related genes, Genetic improvement

## Abstract

**Background:**

The finger lime (*Citrus australasica*), one of six Australian endemic citrus species shows a high natural phenotypic diversity and novel characteristics. The wide variation and unique horticultural features have made this lime an attractive candidate for domestication. Currently no haplotype resolved genome is available for this species. Here we present a high quality, haplotype-resolved reference genome for this species using PacBio HiFi and Hi-C sequencing.

**Results:**

Hifiasm assembly and SALSA scaffolding resulted in a collapsed genome size of 344.2 Mb and 321.1 Mb and 323.2 Mb size for the two haplotypes. The nine pseudochromosomes of the collapsed genome had an N50 of 35.2 Mb, 99.1% genome assembly completeness and 98.9% gene annotation completeness (BUSCO). A total of 41,304 genes were predicted in the nuclear genome. Comparison with *C. australis* revealed that 13,661 genes in pseudochromosomes were unique in *C. australasica*. These were mainly involved in plant-pathogen interactions, stress response, cellular metabolic and developmental processes, and signal transduction. The two genomes showed a syntenic arrangement at the chromosome level with large structural rearrangements in some chromosomes. Genetic variation among five *C. australasica* cultivars was analysed. Genes related to defense, synthesis of volatile compounds and red/yellow coloration were identified in the genome. A major expansion of genes encoding thylakoid curvature proteins was found in the *C. australasica* genome.

**Conclusions:**

The genome of *C. australasica* present in this study is of high quality and contiguity. This genome helps deepen our understanding of citrus evolution and reveals disease resistance and quality related genes with potential to accelerate the genetic improvement of citrus.

**Supplementary Information:**

The online version contains supplementary material available at 10.1186/s12870-024-04988-8.

## Background

Citrus are among the most valued fruits and are cultivated in more than 140 countries in the world [1]. There are six wild citrus species which are endemic to Australia. *Citrus australasica* F.Muell (Australian finger lime) is naturally found in southeast Queensland and northern New South Wales and commercially grown in a number of countries around the world. *C. australasica* is unique among the citrus with finger shaped cylindrical fruits with a distinct pulp which resembles a caviar [2]. The acidic fruit are often used to prepare jams, sauces and drinks, and dried peel is being used as a spice [3]. A high natural phenotypic diversity has been recorded for this species with over 65 cultivars described from the wild [4]. Many cultivars are also being developed by hybridization of *C. australasica* with other citrus species [3] and collectively these accessions have been vegetatively propagated by grafting onto a range of different rootstocks [5]. These accessions are diverse in skin colour [2], volatile constituents [6], phytochemical composition of the fruits [7], taste and seediness of the fruits, and size and shape of the trees [5]. Sexual compatibility between *C. australasica* and other citrus has led to the development of hybrids such as Blood lime (*C. limonia* Osbeck X *C. australasica*), Sunrise lime [(*C. australasica* X *Fortunella* sp. (Champ. ex Benth.) Swingle) X *C. reticulata*)], Sydney hybrid (*C. australasica* X *C. australis* Planch.), Faustrime [*C. australasica* X (*C. aurantifolia* (Christm.) Swingle X *F. japonica* (Thunb.) Swingle))], and Minnie Finger Lime (*C. inodora* F.M. Bailey X *C. australasica*) [8, 9].

Huanglongbing (HLB) disease is one of the most devastating diseases causing severe yield reductions and economic impacts to citrus industries [10]. The disease has caused large scale disruptions in citrus industries in more than 40 countries around the world including those in Asia, Africa and all citrus growing states in the United States [11, 12]. HLB is caused by three species of closely related bacteria: *Candidatus liberibacter asiaticus* (CLas), *C. liberibacter africanus* (Claf) and *C. liberibacter americanus* (CLam), with the first strain of these species being the most destructive and widespread around the world. The disease is transmitted by an insect vector; Asian citrus psyllid (*Diaphorina citri*) [13]. Most commercial cultivars are susceptible to the disease, resulting in reduced yield and a deterioration of quality [14, 15]. Tolerance to HLB disease has been identified in a limited number of citrus accessions and related genera including some citrus cultivars with citrons as a parent [16], Sugar Belle mandarin hybrid [17], *C. maxima* (Burm.) (pummelo) [18], *C. hystrix* DC. (Kaffir lime) [19], *C. latipes* Swingle (khasi papeda) [20], *C. Cavalerie* H. Lev. ex *Cavalerie* ‘2586’ [21], *C. trifoliata* L. (Syn. *P. Trifoliata*) [22], and some accessions of *Murraya paniculata* (L.) [23]. Australian wild limes such as *C. australasica*, *C. australis*, *C. glauca* (Lindlay) Burkill, *C. inodora* and their hybrids, have been reported with tolerance/resistance/partial resistance to this disease [15, 23–26].

High quality reference genomes provide a key resource to better understand the genetics underpinning complex plant agronomical/physiological traits. Here we present the high quality, haplotype resolved genome of *C. australasica*, assembled with PacBio Hifi reads, and Hi-C reads and further manually curated based on our previously assembled *C. australis* genome. The structural and functional characterization of this *C. australasica* genome was used to explore important defense related genes, the genetic variation present within five cultivars of the species, and for comparative genomic analysis against the haplotype resolved genome of *C. australis* [27]. The genome presented in this study should help to accelerate the genetic improvement of citrus by providing a valuable foundation to study genetic resistance to HLB, better understand the genomic diversity present within the species, and examine comparative biology and evolutionary relationships with other citrus species.

## Methods

### Sample collection, DNA and RNA extraction and sequencing, Hi-C sequencing

Young fresh immature leaves of five cultivars of *Citrus australasica* (cultivar names and their morphological characteristics are given in Table S1) were collected from Herbalistics Pty Ltd private orchard located in Maroochy River, Queensland, Australia. Cultivar 2 (Rainbow) was used for genome assembly and other four cultivars were used for variant analysis. Citrus species reported in this study were given their botanical authorities according to Mabberly, 2022 [28]. Genomic DNA was extracted from pulverized leaf tissues using a CTAB (Cetyltrimethyl ammonium bromide) DNA extraction protocol [29]. HiFi reads from Rainbow were generated from two PacBio Sequel II SMRT cells at The Australian Genome Research Facility (AGRF), The University of Queensland, Australia. Total RNA from Rainbow was extracted from pulverized leaves using Trizol and Qiagen kit methods [30] and was sequenced at the AGRF, The University of Queensland, Australia. Snap frozen fresh young leaves from the same individual were sent for Hi-C sequencing at The Ramaciotti Centre for Genomics, University of New South Wales, Australia. The Hi-C library preparation was done using Phase Genomics Proximo Plant Hi-C version 4.0. Illumina sequencing for other four cultivars were performed at AGRF, Victorian comprehensive cancer centre, Melbourne.

### Genome assembly and annotation

Genome assembly was performed with PacBio high fidelity (HiFi) reads and Hi-C reads using the Hifiasm Denovo assembler [31]. Detailed assembly pipeline with different parameters in HiFiasm algorithm can be referred in our previous publication [27]. Contig assemblies generated by hifiasm was scaffolded by Hi-C data using three aligners [Bowtie2 [32], Chromap [33], and BWA [34]], and three latest scaffolding techniques [SALSA [35], YaHS [36], pin_hic [37]. The details for scaffolding are given in Figure S1 and Table S2 and Method S1. The BWA aligner + Arima mapping (https://github.com/ArimaGenomics/mapping_pipeline) + SALSA scaffolding pipeline was selected as the final assembly based on the high contiguity, and the presence of telomeres in scaffolds. In this pipeline, Hi-C reads were first mapped to the Hi-C integrated Hifiasm draft assembly with BWA aligner using Arima-HiC mapping pipeline. BWA first built an index of the contig assembly. Read pairs generated from sequencing were first independently aligned to the reference genome (as single-ends) using BWA-MEM using an end-to-end algorithm. Then 5’-side of the chimeric reads were filtered using filter five end.pl script. After filtering, the filtered single-end Hi-C reads were paired using “two_read_bam_combiner.pl,” which output a sorted, mapping quality filtered, paired-end BAM file for each lane of the sequencing. Read groups were added to the BAM file using Picard tools. Then the paired-end BAM files that were sequenced via two Illumina lanes from the same library were merged and PCR duplicated were removed using Picard tools. The final BAM file was converted to a BED file using bamToBed command from the Bedtools package and was sorted to be used by SALSA. SALSA was used in -e option by specifying the restriction site for the Sau3AI/DpnII endonucleases (GATC). Assigning of contigs to pseudo chromosomes were further supported by manual curation as explained in Table S3.

Genome assembly and annotation completeness was determined using BUSCO in viridiplantae lineage (BUSCO v5.2.2) [38] and the contiguity was assessed using QUAST (version 5.0.2) [39]. Scaffolds were aligned with the previously published genome, *C. australis* [27] to assign them to pseudo chromosomes using D-Genies v.1.4 [40]. The telomeres in pseudochromosomes were identified manually and by telomere identification toolkit (tidk) (https://github.com/tolkit/telomeric-identifier). Ribosomal RNA gene repeats (5s/5.8s/18s/28S rRNA) and satellite repeats at the ends of the scaffolds were detected by nucleotide BLAST in NCBI. The K-mer analysis was performed using Jellyfish (v2.2.10) [41] and Genomescope [42]. Repeat elements in the genome were de novo detected by Repeatmodeler2 version 2.0.1 [43] followed by soft masking by Repeatmasker version 4.0.9_p2 [44]. Quality and adapter trimmed RNA-seq reads were aligned to the soft masked genome using HISAT2 [45]. Structural and functional annotations were performed as mentioned in our previous publication [27]. Gene prediction was performed using Braker3 (https://github.com/Gaius-Augustus/BRAKER).

### Structural and functional differences between *C. australasica* and *C. australis* genomes

Orthologous gene clusters enriched in *C. australasica* and *C. australis* were identified using Orthofinder algorithm incorporated in Orthovenn3 [46]. Pairwise sequence similarities among the longest protein isoform of each protein coding gene of the two species were calculated with an e-value cut off of 1e − 5. The structure of orthologous clusters was defined with an inflation value of 1.5. Unique and shared gene families in *C. australasica* collapsed and haplotype genomes were identified using the above same parameters. The biological processes and molecular functions associated with unique genes were retrieved from combined graph module and the associated pathways were identified from KEGG analysis in OmicsBox 3.0.30. The structural and sequence differences between the two soft-masked genomes of *C. australis* and *C. australasica* at the whole genome level were predicted using Synteny and Rearrangement Identifier (SyRI) [47]. The whole genome alignments were conducted with nucmer with --maxmatch to get all alignments between the two genomes including the -c 100 -b 500 -l 50 parameters. The alignments were filtered using the delta-filter tool and subsequently converted to tab-delimited files using the show-coords command. Syri was used with default parameters and the genomic structures predicted by syri was plotted by plotsr.

### The variant analysis for five different *C. australasica* cultivars

All analysis was undertaken using CLC Genomics Workbench v23,0.4 (Qiagen, USA). Illumina reads from the five cultivars (Table S1) were quality trimmed at 0.01 quality limit (Phred score of 20 and above). They were mapped to the chromosome level assembly of an unmasked reference genome of Rainbow. The reads were mapped using the “map reads to reference” algorithm and the mapping options of Match score (1), Mismatch cost (2), Linear gap cost [Insertion cost (3), Deletion cost (3)], Auto-detect paired distances – yes, Non-specific match handling – map randomly. Mapping was performed at four different mapping stringencies (Figure S2) to select the best mapping setting for the read alignment based on the mapping percentage. The mapping setting of Length fraction of 0.9 and similarity fraction of 0.9 were used as the optimal mapping setting for all the read alignments. The duplicated reads derived from PCR amplification during the sequencing library preparation were removed from the read mappings to avoid creating false positive SNPs in subsequent variant analysis. Structural variant analysis was performed to identify erroneous variants involving insertions, deletions, inversions, translocations, and tandem duplications. Then the local realignment was performed to improve the alignments of the reads in the initial read mapping typically around the erroneous INDEL regions with respect to the reference. The read mappings were then subjected to Fixed ploidy variant detection (FPVD) tool using five minimum frequencies (MF) (Figure S3) and 20% MF was selected as the optimal MF to capture the highest number of single nucleotide variant positions (SNVs). FPVD was performed at the settings of minimum coverage (10), minimum count (2) and minimum frequency (%) (20%). The number of homozygous SNVs (homozygous positions) filtered at 100% variant frequency and the number of heterozygous SNV positions with the frequency ranged between 20 − 80% for the two alleles were calculated in all the cultivars in the whole genome and in the CDS regions with respect to the reference Rainbow genome. The heterozygosity for each cultivar was determined by heterozygous SNV positions as a percentage of Rainbow genome size (344 Mb; estimated by genome assembly).

### The identification of genes related to metabolic pathways and defense in *C. australasica*

The key genes involved in the biosynthesis of terpenoids, and anthocyanins were identified by KEGG pathway analysis [48] using Omics Box 3.0.30 and were further verified by BLAST homology search of other citrus species with an e-value of 1.0E-5, in CLC Genomics Workbench 23.0.4 as mentioned in our previous publication [27]. The antimicrobial proteins were identified based on functional annotation BLAST descriptions and stable antimicrobial peptide (SAMP) homologs were detected by BLAST homology searches against the published SAMP sequence of *C. australasica* [26]. The variations between the gene sequences and the corresponding amino acid sequences of SAMP of *C. australasica* were identified using Clone Manager Ver. 9. The whole genome short reads of *C. australasica* cultivars were mapped to the SAMP homolog of Rainbow genome in CLC using the map reads to reference option with mapping settings of Match score (1), Mismatch cost (2), Linear gap cost [Insertion cost (3), Deletion cost (3)], Length fraction of 0.9 and similarity fraction of 0.9. Defense related genes were mapped onto chromosomes using shinyCircos-V2.0 [49]. Collinear genes in *C. australasica* genome were identified using MCScanX toolkit. For MCScanX, the homology was first searched using the longest isoform of protein coding genes using BLASTP with an e-value threshold of 10^− 10^ [50]. The collinear file was then trans-formatted for micro-synteny view using TBtools [51].

## Results

### Chromosome scale genome assembly of *C. australasica*

Hifi reads with Q33 median read quality were generated from two PacBio SMRT cells yielding 39 Gb (115X) and 37 Gb (108X) of sequence respectively. Hi-C paired end Illumina reads generated from two lanes yielded a total of 768 M reads with a total of 116 Gb data. Hifiasm was run with two options; Hifi reads only and the Hi-C integrated (Hifi reads + Hi-C reads) option (Table S4). The Hifi-reads-only assembly yielded a slightly better contig assembly with a total size of 407 Mb (1,569 contigs), having a 99.3% complete BUSCO and an N50 of 31.7 Mb. The Hi-C integrated Hifi assembly yielded a collapsed assembly with 2,224 contigs (with a total size of 436 Mb), a 99.1% complete BUSCO and an N50 of 31.4 Mb. The two assemblies were independently subjected to scaffolding with Hi-C data.

Scaffolding was performed with Hi-C data using three scaffolding tools using two different pipelines (Figure S1). The results were compared by checking the telomeres at the ends of the scaffolds, the N50 for the whole assembly and by aligning the scaffold assemblies with the previously published genome of *C. australis* [27] (Table S2a-S2g). The Hi-C integrated assembly, assembled by BWA + Arima mapping and the SALSA pipeline was selected as the final best assembly based on the high contiguity, completeness, and the presence of telomeric repeats at the ends of the scaffolds (Method S1, Table S2e). The scaffolds that could be assigned to chromosome level based on the alignments with *C. australis* are shown in Table S2e. The scaffold assembly generated a total of 2,208 scaffolds (with a total length of 436 Mb) with a 99.1% complete BUSCO and an N50 of 33.5 Mb (Table S5).

The top eleven longest scaffolds in the collapsed genome were selected to represent the nine pseudochromosomes as they had the same total BUSCO score (99.1%) as the whole assembly and were anchored to pseudochromosomes by aligning with *C. australis* genome. (Table S5). The pseudochromosomes were labeled as Chr1-Chr9 based on the order of *C. australis* genome. The orientations of the *C. australasica* chromosomes were determined based on those of *C. australis* chromosomes (Figure S4a). Seven pseudochromosomes were composed of one contig. Chr8 was composed of two contigs which were joined by Hi-C. Chr4 was generated by manually joining three scaffolds (manual adjustments) (Table S3). Four pseudochromosomes had telomeres at both terminals, three pseudochromosomes had telomeres at one terminal, one pseudochromosome had one telomere at one end and the other at the peri-terminal region, and one pseudochromosome with no telomeric sequences at either end (Figure S4c). The N50 of the chromosome scale assembly was 35.2 Mb (Table S5). There were some scaffolds (SC9, SC10, SC8, SC11 and SC20) with 5.8 S and 28 S rRNA gene repeats at the terminal regions and some scaffolds (SC5, SC9) with high copy number tandem arrays of satellite DNA repeats at their terminal regions (Table S3).

Dotplots of scaffolds vs. contig assemblies revealed that some medium sized scaffolds (5 Mb – 1.4 Mb) might belong to the nuclear genome (Figure S4b). Those scaffolds and two other small scaffolds (0.85 Mb and 0.7 Mb) with telomeres at the terminal regions were included in the final assembly as unplaced scaffolds as they might be parts of the nuclear genome The assembly containing nine pseudochromosomes and the unplaced scaffolds, totaling 344.2 Mb, is henceforth referred to as the “nuclear genome” of *C. australasica*. The alignment of scaffolds with the *C. australasica* chloroplast genome revealed the sites of insertion of chloroplast fragments in the nuclear genome (Figure S5a and S5b). Some scaffolds smaller than 1.4 Mb might belong to the chloroplast genome (Figure S5c) and were excluded from the nuclear genome assembly. The heterozygosity of the genome was estimated as 1.28% by K-mer analysis.

The two haplotypes were also assembled using the same pipeline and some manual adjustments were done based on their homology with the collapsed genome. The orientation and chromosome numbers of the chromosome scale pseudomolecules were determined with respect to the collapsed assembly (Figure S6, Table S3). The two haplotypes had 98.9% and 99% BUSCO and an N50 of 32.7 Mb and 34.4 Mb for hap1 and hap2 respectively.

### Structural annotation of *C. australasica* genome

The genome was annotated for repeat elements, and protein coding genes. A large portion of the collapsed genome was covered by interspersed repeats (54.6%) with 34.5% unclassified repeats, 2.64% DNA transposons and 17.5% retro transposons. LTR elements were the dominant type of retroelements where Copia and Gypsy elements were present in equal proportions (6.78%) and Pao elements were found in a very small percentage (0.03%). The other types of repeat elements such as rolling circles (0.42%), small RNA repeats (0.67%), satellite repeats (0.39%), simple repeats (0.97%) and low complexity repeats (0.2%) were found in small proportions in the whole nuclear genome (Table S6). RNA-seq trimmed reads (320 million reads, 44 Gb representing 129X coverage of the genome) were used for gene prediction. A total of 36,597 genes were predicted in nine pseudochromosomes of the collapsed genome while 30,050 and 34,139 genes were found for the hap1 and hap2 nine pseudochromosomes respectively (Table 1). The annotation statistics of the nuclear genomes (including the unplaced scaffolds) are given in the Table S7.

**Table 1:** The size of the pseudochromosomes and the number of genes in collapsed, hap1 and hap2 genomes

Chromosome	collapsed		hap1		hap2	
	Size (Mb)	Genes	Size (Mb)	Genes	Size (Mb)	Genes
1	31.4	3,514	31	3,299	30.6	3,489
2	35	4,272	32	3,334	34.4	4,274
3	36.9	3,306	36.6	3,222	35.1	3,095
4	43.9	4,144	39.8	4,366	34.4	3,415
5	49.5	6,541	47.4	4,586	48.9	5,653
6	26.1	2,501	26	2,475	26	2,432
7	33.5	3,830	32.7	3,556	36.5	5,018
8	35.2	4,866	29.1	2,602	34.2	4,219
9	34.8	3,623	29.6	2,610	30	2,544
Total size (Mb)	326.3		304.1		310.1	
**Total genes**	**36,597**		**30,050**		**34,139**	
Annotation completeness (BUSCO)	98.9%		98.8%		99.2%	

### Functional annotation of *C. australasica* genome

The CDS sequences of the collapsed genome (45,935) and the two haplotypes (39,651 of hap1 and 41,516 of hap2) were independently annotated obtaining BLAST hits and GO terms associated with the CDS sequences. BLAST hits were obtained for 40,105 CDS sequences in the collapsed genome and for 35,286 and 39,395 sequences in hap1 and hap2 genomes respectively. Of the sequences with BLAST hits, 21,104 CDS in the collapsed genome, 20,899 CDS in the hap1 genome and 20,966 CDS in the hap2 genome underwent GO mapping and annotation. The majority of the CDS sequences of the collapsed and the two haplotypes received BLAST hits from other citrus species. The highest number of sequences received BLAST hits from *C. sinensis* (179,114 - collapsed genome, 166,574 - hap1 genome, and 175,964 - hap2 genome), followed by *C. clementina* (39,949 - collapsed genome, 39,304 - hap1 genome, 39,605 - hap2 genome) and *C. unshiu* (27,052 - collapsed genome, 26,723 - hap1 genome, 26,784 - hap2 genome) and a small percentage of sequences from other species (Figure S7a, S7b, S7c). Sequences annotated with IPS, their families distribution, GO-levels and enzyme codes of the collapsed genome are shown in Figure S7d, S7e, S7f. The coding potential assessment of the CDS sequences in the collapsed genome with no BLAST hits (5,830) revealed 99.8% and 99.1% CDS with coding potential based on the models from coding and non-coding sequences of *Arabidopsis thaliana* and citrus respectively. The coding potential of the sequences with no BLAST hits for the two haplotypes also indicated a high number with coding potential (Figure S8a, S8b, S8c, S8d).

### Structural and functional comparison between *C. australasica* and *C. australis* genomes and among *C. australasica* assemblies

**Fig. 1:**
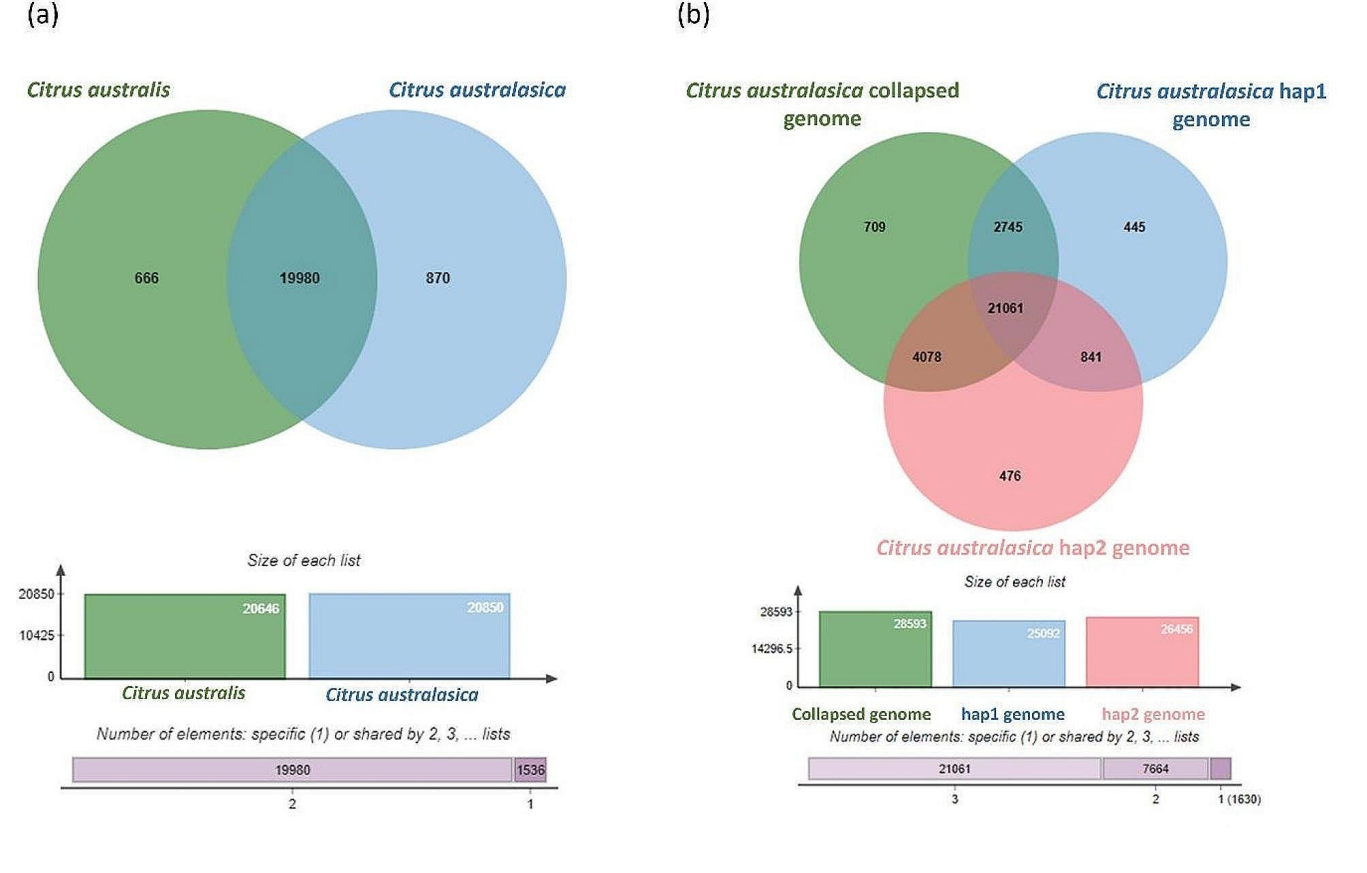
The orthologous gene clusters in *C. australasica* and *C. australis* genomes identified using Orthovenn3. **(a)** The orthologous gene clusters present in *C. australis* and *C. australasica*. 19,980 orthologous clusters (48,185 genes) were shared between the two species and 666 gene clusters (4,191 genes) and 870 gene clusters (12,748 genes) were unique to *C. australis* and *C. australasica* respectively. **(b)** Orthologous gene clusters among *C. australasica* collapsed genome and the two haplotype genomes. 21,061 gene clusters (76,167 genes) were shared by three genomes and 709 clusters (3,307 genes), 445 clusters (1,676 genes), and 476 clusters (1,696 genes) were specific to the collapsed, hap1 and hap2 assemblies

The inference of orthologs from orthovenn3 revealed 19,980 shared orthologous clusters between *C. australasica* and *C. australis* corresponding to 48,185 shared genes. The number of unique orthologous clusters (gene families) in *C. australasica* (870) were higher than *C. australis* (666) (Fig. 1a). The 870 unique orthologous clusters of *C. australasica* included 12,748 unique protein coding genes and the 666 unique orthologous clusters of *C. australis* contained 4,191 unique protein coding genes. In addition to the unique orthologous clusters, there were 4,487 singletons in *C. australasica* and 5,566 singletons in *C. australis,* which had no orthologous genes identified in the other species and they could not be assigned to any cluster within the species. Therefore, the genes in unique orthologous clusters and singletons are henceforth referred to as unique genes in each species. Of them, 13,661 unique genes of *C. australasica* and 8,121 unique genes in *C. australis* were within their nine chromosomes.

The functional analysis of the unique genes of *C. australasica* revealed that they were enriched in biological processes including stress response, protein modification, cellular component organization, response to organic substance, transport and regulation of gene expression with molecular functions such as nucleic acid binding, hydrolase activity, protein binding, catalytic activities, ATP binding, oxidoreductase activity and transition metol ion binding. KEGG pathway analysis showed that the unique gene sequences of *C. australasica* were primarily involved in purine metabolism (155), thiamine metabolism (145), plant-pathogen interactions (92) (Table S8) (out of the total 345 genes related to plant-pathogen interactions in *C. australsica*, 92 genes were unique), phenylpropanoid biosynthesis (55), diterpenoid biosynthesis (48), and Tryptophan metabolism (44) (Figure S9). The 92 unique genes associated with plant-pathogen interactions encode disease resistance proteins [NB-ARC domain containing R proteins, nucleotide binding and leucine rich repeat proteins (NLRs), leucine-rich repeat receptor-like protein kinases (LRR-RLKs)], calcium sensor proteins [calcium-dependent protein kinases (CPDKs), calmodulin (CaM) and calmodulin-like proteins (CMLs)], retrovirus-related pol polyproteins, glycerol kinases, pathogenesis-related protein 1, cyclic nucleotide-gated ion channels, and many other hypothetical proteins. The functional analysis of the *C. australis* unique protein clusters indicated that they were associated with many biological processes including protein modification, cellular component organization, transport, defense response, phosphate containing compound metabolic process. The KEGG analysis showed that the unique genes in *C. australis* were mainly involved in purine metabolism (80), plant pathogen interactions (76) and Thiamine metabolism (75) (Figure S10).

The orthologs comparison among the three assemblies of *C. australasica* revealed 3,307 genes unique to collapsed genome, 1,676 unique to hap1 genome and 1,696 genes unique to hap2 genome which are in clusters as depicted in Fig. 1b. In addition, there were 2,155, 1,610, 1,471 singletons identified in the collapsed, hap1 and hap2 assemblies respectively. There were 21,061 shared gene families containing 76,167 shared genes among the three assemblies. There were 13,801 genes (4,078 gene families) shared between collapsed and hap2 assemblies. 8,667 genes (2,745 gene families) were shared between the collapsed and hap1 assembly and 2,830 genes (841 gene families) were shared between the two haplotypes which were not present in the collapsed assembly. Functional characterization of the collapsed and haplotype assemblies specific genes revealed that they were associated with many different cellular, metabolic, biosynthetic processes, and stress responses (Figure S11).

**Fig. 2:**
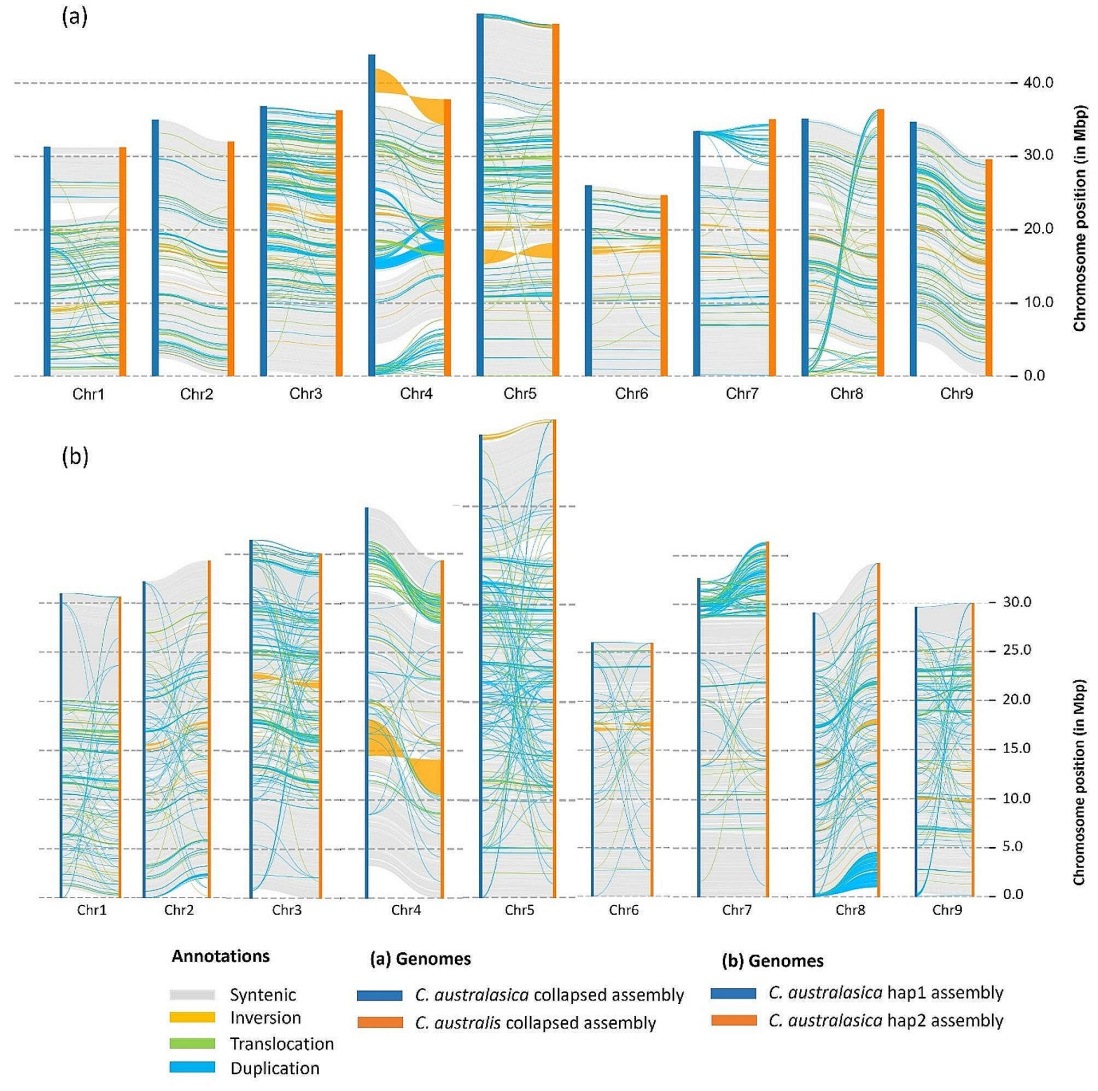
Structural genomic differences between *C. australasica* and *C. australis* genomes and *C. australasica* haplotypes. Syntenic regions are indicated in grey color and unaligned regions are shown in white color. Different types of rearranged regions are shown with respective color codes. The analysis was done using Synteny and Rearrangement Identifier (SyRI) **(a)** The structural comparison between *C. australasica* and *C. australis* collapsed assemblies **(b)** The structural differences between *C. australasica* haplotypes

Structural and local sequence variations between *C. australasica* and *C. australis* genomes revealed the conserved and rearranged regions of the two genomes (Fig. 2a). Large inversions were found in Chr4 and Chr5 and small inversions were found in all chromosomes. Translocations and duplications were found in all chromosomes and large-scale translocations and duplications were found in Chr3, Chr4, Chr5, Chr7 and Chr8. A relatively smaller number of rearranged regions were found in Chr6, which was the smallest chromosome in both the species. Local sequence variations such as SNPs (5,437,677), insertions (542,557), deletions (445,565), highly diverged regions (4,915) and tandem repeats (137) were annotated in both syntenic and rearranged regions of the two genomes with the help of whole genome alignments (Fig. 2a). The two haplotype assemblies of *C. australasica* were found to have many structural variations across the nine pseudochromosomes (Fig. 2b). A large inversion was present in chr4, and small-scale inversions were found in all chromosomes. Translocations were prominent in chr4, and duplications were prominent in Chr5, Chr7 and Chr8.

### Genetic diversity in different *C. australasica* cultivars

The genetic diversity within *C. australasica* was determined using five cultivars, including Rainbow for which the genome was assembled in the present study. Illumina reads for the five cultivars (between 9 and 13 Gb data with coverage ranging from 28X – 39X genome size of *C. australasica*) were used in the fix ploidy variant analysis using Rainbow as the reference (Table S9). Different variant types (insertions, deletions, single and multi-nucleotide variants, and replacements) ranging between 1.9 M and 3.5 M were found for the five cultivars where many of them were SNVs. At the whole genome level, the total number of SNV positions which include heterozygous SNVs and homozygous SNVs (at 100% variant frequency) were in the range between 1.6 M and 2.8 M (Figure S12). The total number of SNV variant positions in CDS regions were in the range between 0.1 M and 0.18 M for the five cultivars. Based on the total SNVs, *C. australasica* cv 3 (Red champagne) and *C. australasica* cv 1 were the most and less divergent cultivars respectively with respect to Rainbow (Figure S12). The heterozygosity estimated based on the Rainbow genome size was the highest for *C. australasica* cv 3 (Red champagne) (0.56) and the lowest for *C. australasica* cv 1 (0.35) (Table 2).

**Table 2:** SNVs and heterozygosity values in five *C. australasica* cultivars with respect to *C. australasica* cv 2 (Rainbow) as the reference

Cultivar		Whole genome				CDS				Heterozygosity
		Heterozygous SNVs	100% homozygous SNVs	Total SNVs		Heterozygous SNVs	100% homozygous SNVs	Total SNVs		
*C. australasica* cv 2 (Rainbow)		1,601,626	37	1,601,663		104,730	1	104,731		0.47
*C. australasica* cv 1		1,192,418	1,037,382	2,229,800		81,382	68,639	150,021		0.35
*C. australasica* cv 4 (Red Finger lime)		1,407,305	1,053,367	2,460,672		95,624	66,596	162,220		0.41
*C. australasica* cv 3 (Red champagne)		1,927,999	842,733	2,770,732		130,940	50,892	181,832		0.56
*C. australasica* cv 5 (Ricks red)		1,860,975	794,928	2,655,903		127,859	49,745	177,604		0.54

Reference genome size 344.2 Mb including nine pseudochromosomes and unplaced scaffolds

### A selected set of important genes in *C. australasica*

#### Disease resistant genes

**Fig. 3:**
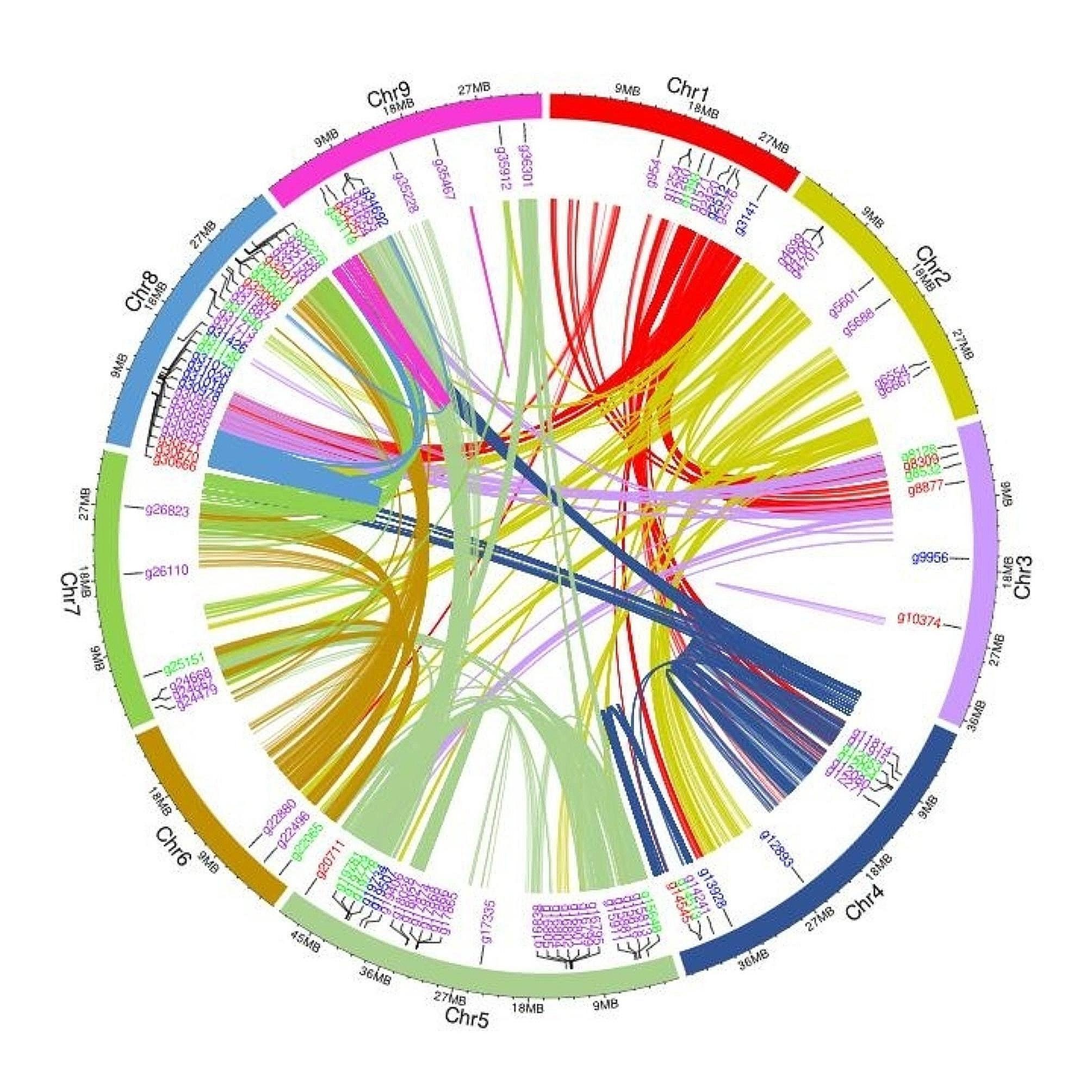
The Circos plot indicating the chromosomal locations of defense related genes in the *C. australasica* genome. Purple indicates the genes encoding leucine rich repeat proteins (LRR), red indicates the genes encoding pathogenesis related (PR) proteins, blue indicates the genes encoding guanine-nucleotide binding proteins and green indicate the genes encoding antimicrobial proteins (stress-responsive A/B barrel domain containing proteins, defensins, thionins, non-specific lipid transfer proteins, snakins, hevein-like proteins, knottin-type proteins). The innermost links indicate the collinear genes within the genome identified by whole genome self-homology and gene location information. Different colors for the links indicate the chromosome of origin of the links. Some collinear genes were identified within the same chromosome while others were identified between chromosomes. Collinear genes represent homologous genes in conserved orders on corresponding chromosomes. Circos plot was generated using shinyCircos-V2.0 and TBtools

A wide array of antimicrobial proteins/peptides (AMPs) were identified through functional annotation in *C. australasica* collapsed genome. Three antimicrobial genes [g22065 (Chr6), g32276 (Chr8), g34118 (Chr9)] coding for peptides containing stress-responsive A/B barrel domain were identified in the collapsed genome (Fig. 3). Of these three genes, g34118 was identified as a homolog to the previously detected short version of stable AMP (SAMP) in HLB resistant citrus species (204 bp) [26]. The gene g34118 was transcribed into three transcripts of 549 bp, 462 bp, and 330 bp. The third transcript having 330 bp (encoding 110 aa) showed the highest homology with a major portion of the 204 bp SAMP sequence (encoding 67 aa) of the previously reported SAMP of *C. australasica* with twelve single nucleotide polymorphisms. Similarly, the SAMP homologs, identified in Chr9 of the two haplotypes (hap1; g27559 of 330 bp and, hap2; g31668 of 330 bp) showed 100% identity with the corresponding collapsed SAMP gene (Figure S13a, S13b). The SAMP identified in the previous study had two cysteine residues, whereas the Rainbow SAMP homologs had no cysteine residues. In addition to the SAMP sequences, two other types of antimicrobial peptides having the stress-responsive A/B barrel domain were identified in the two haplotypes similar to the collapsed genome (Table S10).

The re-annotation of the *C. australis* genome [27] using Braker3 identified one SAMP homologous gene encoding two transcripts which are longer than those of *C. australasica*. The alignment of these two transcripts with *C. australasica* SAMP sequences showed a homology with them, however it was not as high as the sequence homology of *C. australasica* SAMP sequences (Figure S14). Furthermore, the alignments of the 110 aa antimicrobial peptides found in Rainbow and other HLB resistant and susceptible citrus species with 67 aa of SAMP sequence of *C. australasica* showed a high aa similarity among all of them (Figure S15).

In addition to stress-response A/B barrel domain-containing protein, other types of AMPs such as defensins, thionins, non-specific lipid transfer proteins, snakins, hevein-like proteins, knottin-type peptides were identified in the genome. Other defense-related genes including cysteine-rich receptor-like protein kinases (CRKs), of which 14 genes encoding CRK 10, one encoding CRK 25 and others encoding other types of CRKs were identified in the genome (Table S11). There were eleven genes for pathogenesis related (PR) proteins of which three were PR-1 proteins (Fig. 3, Table S12). In addition, the annotation identified 61 leucine rich repeat proteins (LRR) genes, 13 guanine nucleotide-binding proteins (Fig. 3, Table S13), 34 glutathione-S-transferase genes, 28 oxoglutarate (2OG) and Fe(II)-dependent oxygenases, 22 cellulose synthase genes, 26 β-1,3-Glucanase genes, and many other genes related to anthocyanins, terpenoids, amino acids (phenylalanine, tyrosine, and tryptophan) and antioxidants (flavonoids, carotenoids, tocopherols) in the Rainbow genome.

#### Volatile compounds encoding genes

KEGG pathway analysis identified 112 genes involved in terpenoid backbone biosynthesis (Table S14), 82 genes for monoterpenoid biosynthesis (including geranyl phosphate, geraniol, linalool, myrcene, limonene, α-terpineol, camphene, neomenthol) (Table S15), 89 genes for diterpenoid biosynthesis (Table S16) and 48 genes for sesquiterpenoid and triterpenoid biosynthesis (α-farnesene, β-farnesene, germacrene, valencene, β-carophyllene, β-amyrin, α-amyrin, α-humulene) (Table S17) in the Rainbow genome.

#### Curvature thylakoid protein genes

A total of 8,314 genes encoding curvature thylakoid proteins (CURT1) which belong to four isoforms were identified in the genome. There was one gene encoding CURT1A on Chr5 with 495 bp, one gene encoding CURT1B in Chr5 having 510 bp and one gene for CURT1C in Chr1 transcribed into two CDS sequences (465 bp and 444 bp). There were 8,311 genes encoding CURT1D proteins (Table S18), with majority of them being identified within the 9 chromosomes. The lowest number of genes (572) were identified in Chr9, and the largest number of genes were identified in Chr8 (1,619) with open reading frames in the CDS sequences. The CURT1D proteins were present as large tandem arrays of gene clusters within the chromosomes with the smallest gene having 252 bp (encoding 84 aa) and the largest gene having 30,726 bp (encoding 10,242 aa). No CURT1 genes were identified in Chr3 and Chr6. In contrast, only 357 CURT1 genes were found in the *C. australis* genome (Table S19).

#### Red/orange coloration related genes

**Fig. 4:**
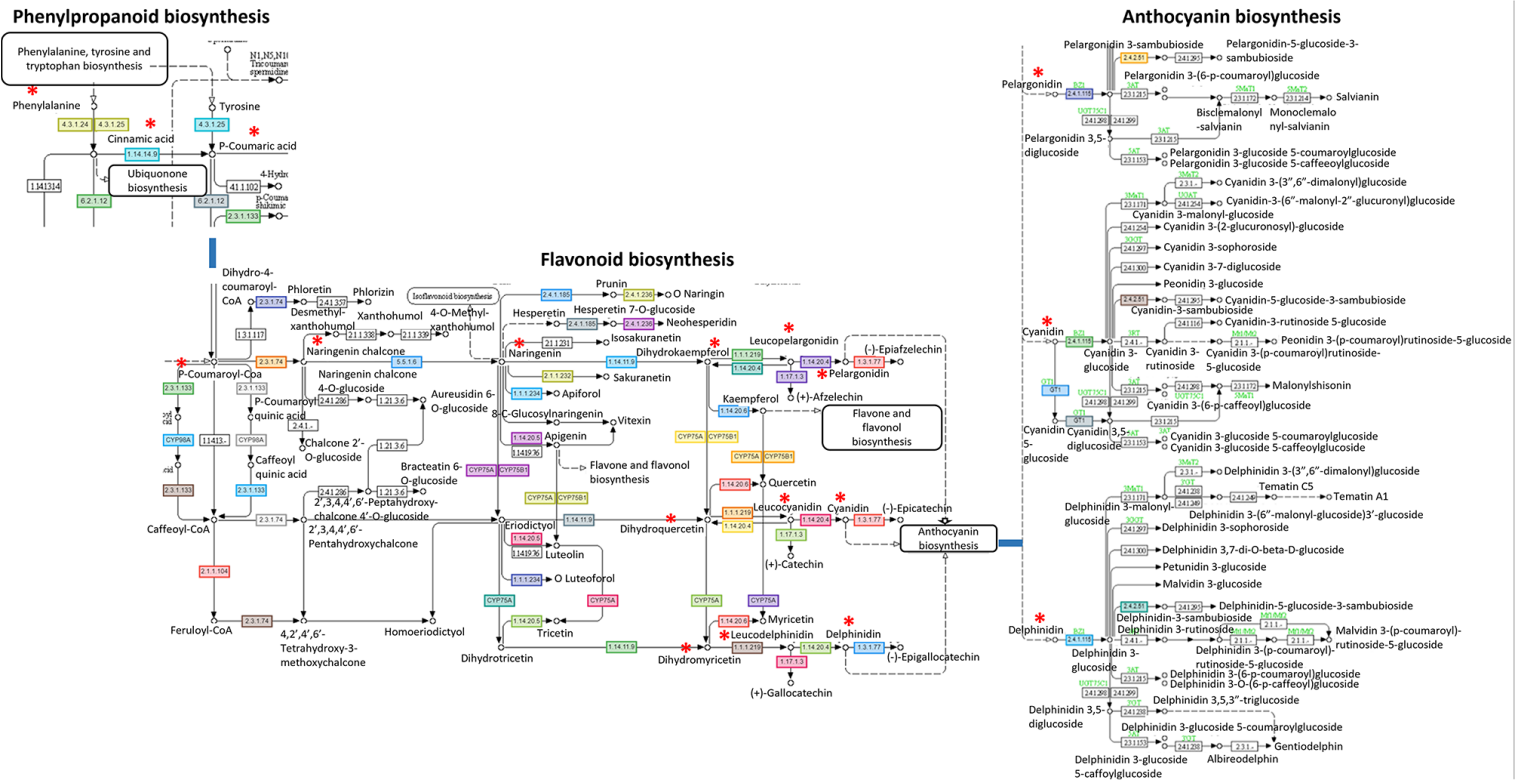
Anthocyanin biosynthetic pathway of *C. australasica* reproduced with permission of Kanehisa Laboratories [48]. The main components in anthocyanin pathway are shown with red asterisks. Phenylalanine undergoes catalysis via a series of steps producing Cinnamic acid, Coumaric acid, 4-Coumaroyl-CoA, Naringenin chalcone, Naringenin, Dihydrokaempferol, Dihydroquercetin, Dihydromyricetin, Leucoanthocyanidins, Anthocyanidins, and Anthocyanins. The major types of anthocyanins identified from the annotation of *C. australasica* by KEGG analysis were pelargonidin, pelargonidin-3-sambubioside, cyanidin, cyanidin 3-glucoside, cyanidin 5-glucoside, cyanidin 3,5-diglucoside, cyanidin-3-sambubioside, delphinidin, delphidin-3-sambubioside, delphinidin 3-glucoside. The pathways were retrieved from KEGG pathway analysis

*C. australasica* cv Rainbow has a red/yellow warty skin and pink colored clear vesicles inside the fruit. Anthocyanins and β-citraurin are the two major pigments involved in the orange-reddish color of citrus fruits. The structural and regulatory genes involved in anthocyanin production were identified in *C. australasica* genome. Structural genes involved in the production of enzymes needed for the biosynthesis of anthocyanins are depicted in Figure S16. A group of upstream genes encoding CHS, CHI, F3H and downstream genes encoding F3’M, F3’5’H, DFR, ANS, UFGT were known to play major roles in pigmentation [52]. The annotation identified 13 CHS genes, 3 CHI genes, 5 F3H genes, 2 F3’M genes, 1 F3’5’H gene, 2 DFR genes, 1 ANS gene, 2 genes of UFGT (Figure S16) (Table S20). A total of 11 genes involved in the biosynthesis of major anthocyanins including pelargonidin, pelargonidin-3-sambubioside, cyanidin, cyanidin 3-glucoside, cyanidin 5-glucoside, cyanidin 3,5-diglucoside, cyanidin-3-sambubioside, delphinidin, delphidin-3-sambubioside, delphinidin 3-glucoside were identified in the genome (Fig. 4).

Four types of regulatory genes are involved in anthocyanin gene expression in plants. There were two Ruby homologs in Chr6, one bHLH (Noemi) homolog on Chr5, five WD-40 protein encoding genes on Chr1, Chr2, Chr4 and Chr5, and 43 WRKY TF genes (Table S21) scattered on all chromosomes in the Rainbow genome.

β-citraurin is encoded by carotenoid cleavage dioxygenase 4 (CCD4) gene. There were two CCD4 genes identified in collapsed, hap1 and hap2 genomes on Chr7 and Chr8 and one additional gene was identified on Chr6 of the hap1 genome (Table S22).

## Discussion

Here, we present the first report of a haplotype resolved chromosome level assembly of *C. australasica*, which is one of the most important endemic limes in Australia. The assembly was produced *de novo* and further manually curated with the help of a previous *C. australis* genome to achieve a more complete chromosome level assembly. With that, 95% of the nuclear genome of the collapsed assembly, 95% of the hap1 genome and 96% of the hap2 genome could be anchored to chromosome level. The high N50s of the final chromosome level assemblies for collapsed (35.2 Mb), hap1 (32.7 Mb) and hap2 (34.4 Mb) indicate high contiguities of the assembled genomes. The high assembly and annotation BUSCO for the collapsed and two haplotypes indicated that the assembled genomes and annotated gene sets had captured most of the single-copy orthologs conserved in the viridiplantae lineage. We also compared different scaffolding pipelines using three recent scaffolding tools (SALSA, YaHS, Pin_hic) and different Hi-C read aligners (bowtie2, chromap, bwa) to select the best pipeline to generate chromosome scale assemblies that were complete, contiguous, and accurate. The accuracy was determined by checking the telomeres, N50s and mapping the scaffolds against the *C. australis* genome. Manual inspection of the assemblies helped the identification of interior telomeres in several instances with the YaHS tool. The joining of the contigs were similar and accurate for SALSA and Pin_hic when those tools were used with BWA aligner to align the Hi-C reads through the Arima mapping pipeline. We proceeded with BWA + Arima mapping + SALSA for scaffolding the collapsed, hap1 and hap2 assemblies. Although this pipeline resulted in accurate assembly for the collapsed genome, it produced some misassembles (contigs were wrongly oriented) for the haplotypes, therefore, some manual curations were done to correct them. A genome for *C. australasica* was recently assembled using Oxford Nanopore Technology (ONT) and Hi-C reads [53]. This genome was not haplotype resolved and the collapsed genome had a contig N50 of only 1.9 Mb. The assembled and phased genome reported in the present study is significantly better in terms of the assembly contiguity, gene completeness and phasing.

Of the nine chromosomes, four pseudochromosomes had telomeres at both ends. The presence of extensive rRNA gene repeats and large tandem arrays of satellite repeats at the terminal regions might be the main reason for not being able to assemble other pseudomolecules as complete chromosomes. Many plant genomes constitute repetitive regions such as transposable elements (DNA TE/ LTR RE), interspersed nuclear elements (SINE/LINE), tandem arrays of satellite repeats and rDNA, which has become a challenge in precisely assembling plant genomes to the chromosome level [54]. Some repetitive DNA sequences are highly conserved among plants whereas some repeat DNA are specific to some genera, species and even to some chromosomes in the same accession [55]. Citrus genomes are also characterized by high repetitive contents mainly at centromeric, pericentromeric, telomeric and sub telomeric regions [56, 57]. In Chr4, the telomeric repeat at one terminal was not at the very end, instead it was found at a sub-telomeric region, and satellite repeats were found next to the telomeric repeat at that terminal. Previous studies have reported the presence of interstitial telomeric repeats (ITRs) at pericentromeric and sub-telomeric regions [58, 59] and it could be possible that *C. australasica* has an ITR at a sub-telomeric region on Chr4.

Gene family clustering analysis revealed 19,980 shared orthologous clusters between *C. australasica* and *C. australis* indicating their conservation in the two species after the species divergence. The corresponding genes of the unique orthologous clusters in each species might have undergone sequence changes over the years of evolution after their divergence from the last common ancestor, and thus have attained new functions. This analysis revealed a high number of unique genes in the *C. australasica* genome when compared to the *C. australis* genome. The high number of unique genes might be due to the large number of total genes annotated in the *C. australasica* genome (7,431 genes more in *C. australasica*). The unique genes in *C. australasica* are primarily involved in resistance to plant pathogens. LRR proteins recognize pathogen effectors and trigger innate immunity in plants [60]. Calcium, which acts as a secondary messenger in plants and its sensors are important in abiotic and biotic stress resistance [61]. CDPKs mediate innate immunity in plants by regulating oxidative burst and hormone signal transduction in response to plant pathogens [62]. Cyclic nucleotide-gated ion channels which regulate the calcium uptake in plants are known to play important roles in stress response, plant immunity and development [63]. Glycerol kinases are also involved in enhancing immune responses in plants [64]. Studies have shown that thiamine related genes can enhance the responses to biotic and abiotic stresses in plants [65, 66].

The differences in the collapsed and haplotype specific genes identified in *C. australasica* might be due to the sequence variations present in the two haplotypes. The unique genes identified in the collapsed genome might be due to the sequence variations between the haplotypes resulting in a combined gene being annotated in the collapsed genome. All chromosomal lengths of the collapsed genome are longer than those of the haplotypes, except in Chr7 where the Chr7 in hap2 is longer than the collapsed and hap1. Some genes in the collapsed and hap2 might have been annotated in those additional regions in the chromosomes resulting of them being identified as unique by the orthovenn3 analysis. The collapsed genome may contain one of the alleles of the two haplotypes in the heterozygous regions of the genome and these genes might be represented in the shared gene families between collapsed and each haplotype. In the homozygous regions of the genome, the collapsed assembly might have picked one of the haplotypes alleles, thus these genes might have shared among the three assemblies. Significant structural variations between haplotypes such as insertions in genes, chromosomal rearrangements, allele specific expressions, and presence and absence variations have been studied extensively in previous research [67] suggesting the importance of phased assemblies in assessing genomic and phenotypic characteristics.

*C. australasica* has a high natural genetic diversity with a large number of VOC in different cultivars having β-citronellol, citronellal, γ-terpinene, and limonene as the predominant constituents [68, 69]. A previous study has shown that *C. australasica* is rich in VOC such as citronellal, nonanal, β-phellandrene, δ-elemene, α-farnesol, β-farnesol which can act as antimicrobial agents to provide the plants with resistance against HLB. *C. australasica* is also known to contain high levels of some amino acids including phenylalanine, tyrosine, and tryptophan and antioxidants which modulate plant responses to pathogens [17]. Many genes related to VOCs, amino acids and antioxidants were identified in the present genome and these might play a role in modulating defense against the HLB causing pathogens.

Plants produce different families of AMPs which are rich in cystine residues and have antibacterial, antifungal, antiviral and anti-parasite activities. They target and rupture the cell membranes of pathogenic organisms resulting in loss of intracellular ions causing cell death [70, 71]. A novel class of short SAMP, having only two cysteine residues, was recently identified from *C. australasica* showing an ability to suppress the growth of CLas and boost the host immunity against further HLB infections [26]. The present annotation identified genes encoding different families of AMPs which have not previously been reported in *C. australasica* which might play important roles in plant immunity against HLB. The gene encoding stress-response A/B barrel domain-containing protein HS1 identified from the collapsed and two haplotypes showed a high sequence homology to previously identified SAMP, however it had a longer sequence (330 bp/110 aa). The 110 bp long version of SAMP sequence is present in both HLB resistant and susceptible citrus species, and they all have a high sequence similarity with a major portion of the short version of the SAMP sequence of *C. australasica*. *C. australis* had a SAMP homologous gene encoding two relatively long peptides which also showed sequence homology with *C. australasica* 67 aa. All this data suggests that both HLB resistant and susceptible citrus species might encode the longer versions of SAMP peptides (large protein precursors), which may then be cleaved into mature polypeptides in resistant cultivars and subjected to further post-translational modifications resulting in short versions of SAMP in resistant cultivars. The other types of defense related genes identified in the genome might have possible roles in HLB resistance as many of those genes have previously been characterized with high expression levels in response to HLB infection in *C. australasica* [15] and other HLB resistant species [22].

The whole genome alignment of *C. australis* and *C. australasica* revealed that the two genomes had a high level of synteny across the 9 chromosomes with some rearrangements in all the chromosomes. These rearranged regions characterized by inversions, translocations and duplications might be the main reason for underlying phenotypic differences between the two species. *C. australis* naturally grows as shrubs or small trees with green, globose, or less-globose fruits and leaf oil predominantly containing α-pinene [72]. *C. australasica* is a thorny tree and grows as understory shrubs or small trees in sub-tropical rainforests [73]. Many CURT1D genes found in the *C. australsica* genome might explained its tolerance to shade within the forest canopy where it occurs. Within the chloroplasts of a plant cell, thylakoids are organized into grana and the thylakoid membranes are the sites where the light reactions of photosynthesis occur. The curving of the thylakoid membranes at the grana margins which is necessary for grana formation is mediated by CURT1 proteins. Previous studies have shown that plants that are adapted to low light have many layers of thylakoid membranes per granum relative to those found in plants adapted to bright sun light, which provides a means of enhancing the photosynthetic efficiency in shade tolerant plants [74, 75]. There is a great variation of the genes with homology to CURT1D identified in the *C. australaisca* genome ranging from 252 bp to 30,726 bp with complete open reading frames, although it is not known whether they are all functional. The previously reported CURT1 genes in citrus also had a variation in size, however, they all had CDS sequences of more than 440 bp. Therefore, it is possible that the smaller CURT1 proteins might be non-functional. This is the first report of CURT1 genes in citrus and further studies are required to understand the expression of these CURT1 genes and their roles in photosynthesis of citrus plants under fluctuating light conditions.

*C. australasica* is unique within the Rutaceae family with finger-shaped fruits, novel caviar-like pulp, unusual organoleptic properties (citronellal/limonene/isomenthone), and wide variation in skin and pulp colours [2, 73]. . The five cultivars used in this study varied in terms of their fruit skin and pulp colours, tree size, seediness and time of flowering. The whole genome variant analysis based on SNVs revealed structural variations among these five different cultivars which might explain some of the variation in their phenotypes. This revealed *C. australasica* cv 1 and cv 4 (Red finger lime) as the closest and *C. australasica* cv 3 (Red champagne) and cv 5 (Ricks Red) as the most divergent cultivars with respect to the Rainbow cultivar. The different skin and pulp colours of the five cultivars might be regulated at the transcription level by anthocyanin regulatory genes [52, 76] and β-citraurin [77]. The structural variations of anthocyanin regulatory genes with their differential expression have been extensively studied among differently coloured citrus types [52, 78]. The red pigments of *C. australasica* are also known to indirectly suppress the CLas infection by impeding the visual signals to *D. citri* and thereby preventing their feeding [17]. The high-quality genome we present here will facilitate the study of gene variations regulating a diverse array of red-orange colours in *C. australasica* in the future, which will provide breeders with direction for developing novel cultivars with high consumer appeal.

## Conclusion

The lack of a high-quality genome for *C. australasica* has greatly hindered genomic research, particularly in relation to HLB resistance. Here we present the first report of a high quality, haplotype resolved genome for *C. australasica* and its structural and functional characterization. An assessment of genetic diversity present within this species and genomic variations and commonalities with *C. australis* at a structural and functional level are also provided. This should prove to be a valuable genomic resource to accelerate molecular breeding for the genetic improvement of citrus and will lay the foundation for comparative genomics to broaden our understanding of this unique species in the citrus genus.

### Electronic supplementary material

Below is the link to the electronic supplementary material.

Supplementary Material 1

Supplementary Material 2

## Data Availability

Raw PacBio HiFi data, and RNA-seq sequence data generated in this study have been deposited in NCBI Sequence Read Archive (SRA) under the BioProject [PRJNA1019815] and Biosample [SAMN37501217] with accession IDs SRR26236521, SRR26251946, and SRR26202756. The whole genome short reads are available under the Bioproject [PRJNA1010857] and the Biosample [SAMN37218318] with accession ID SRR25915022. The whole genome sequence data reported in this paper have been deposited in the Genome Warehouse in National Genomics Data Center [79, 80] Beijing Institute of Genomics, Chinese Academy of Sciences / China National Center for Bioinformation, under accession numbers GWHDUCH00000000 (collapsed genome), GWHDUEL00000000 (hap1 genome), GWHDUEM00000000 (hap2 genome), BioProject [PRJCA019902], and Biosample [SAMC3069019] that is publicly accessible at https://ngdc.cncb.ac.cn/gwh.

## Abbreviations

HLB: Huanglongbing
Clas: *Candidatus liberibacter asiaticus*
Claf: *C. liberibacter africanus*
Clam: *C. liberibacter americanus*
CTAB: Cetyltrimethyl ammonium bromide
HiFi: PacBio high fidelity
FPVD: Fixed ploidy variant detection
MF: Minimum frequencies
SNVs: Single nucleotide variant positions
NLRs: Nucleotide binding and leucine rich repeat proteins
LRR-RLKs: Leucine rich repeat receptor-like protein kinases
CPDKs: Calcium dependent protein kinases
CaM: Calmodulin
CMLs: Calmodulin like proteins
SyRI: Synteny and Rearrangement Identifier
AMPs: Antimicrobial proteins/peptides
SAMP: Stable AMP
CRKs: Cysteine rich receptor-like protein kinases
PR: Pathogenesis related
LRR: Leucine rich repeat proteins
CURT1: Curvature thylakoid proteins
CCD4: Carotenoid cleavage dioxygenase 4
ONT: Oxford Nanopore Technology
ITRs: Interstitial telomeric repeats
