# Supplementary material for: The genome of Citrus australasica reveals disease resistance and other species specific genes
Source: BMC Plant Biol. 2024 Apr 10;24:260. doi: 10.1186/s12870-024-04988-8 (PMC11005238; doi:10.1186/s12870-024-04988-8)
Supplement: Supplementary file 1 — Supplementary Material 1 [file 12870_2024_4988_MOESM1_ESM.docx]

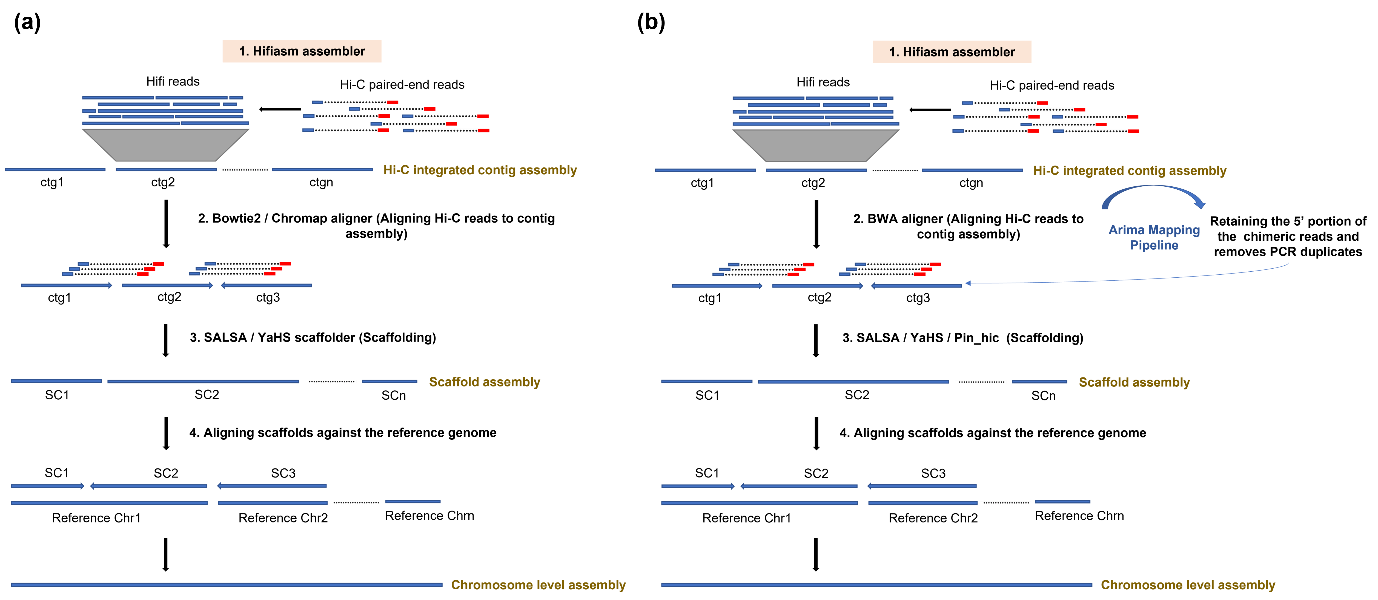


**Figure S1** The genome scaffolding using two different pipelines (a) In the first pipeline, the Hi-C reads were aligned to the Hi-C integrated contig assembly using Bowtie2 and Chromap aligners independently and was subjected to SALSA and YaHS scaffolders for scaffolding. The output scaffolds were further aligned against the reference genome (*C. australis*) to generate chromosome level assemblies. (b) In the second pipeline, the Hi-C reads were mapped to the contig assembly using BWA aligner and were subjected to Arima mapping pipeline to retain only the 5’ portions of the chimeric reads and to remove the PCR duplicates followed by scaffolding using SALSA, YaHS and Pin_hic tools. The output scaffolds were aligned with the reference (*C. australis*) to generate the chromosome scale assemblies.


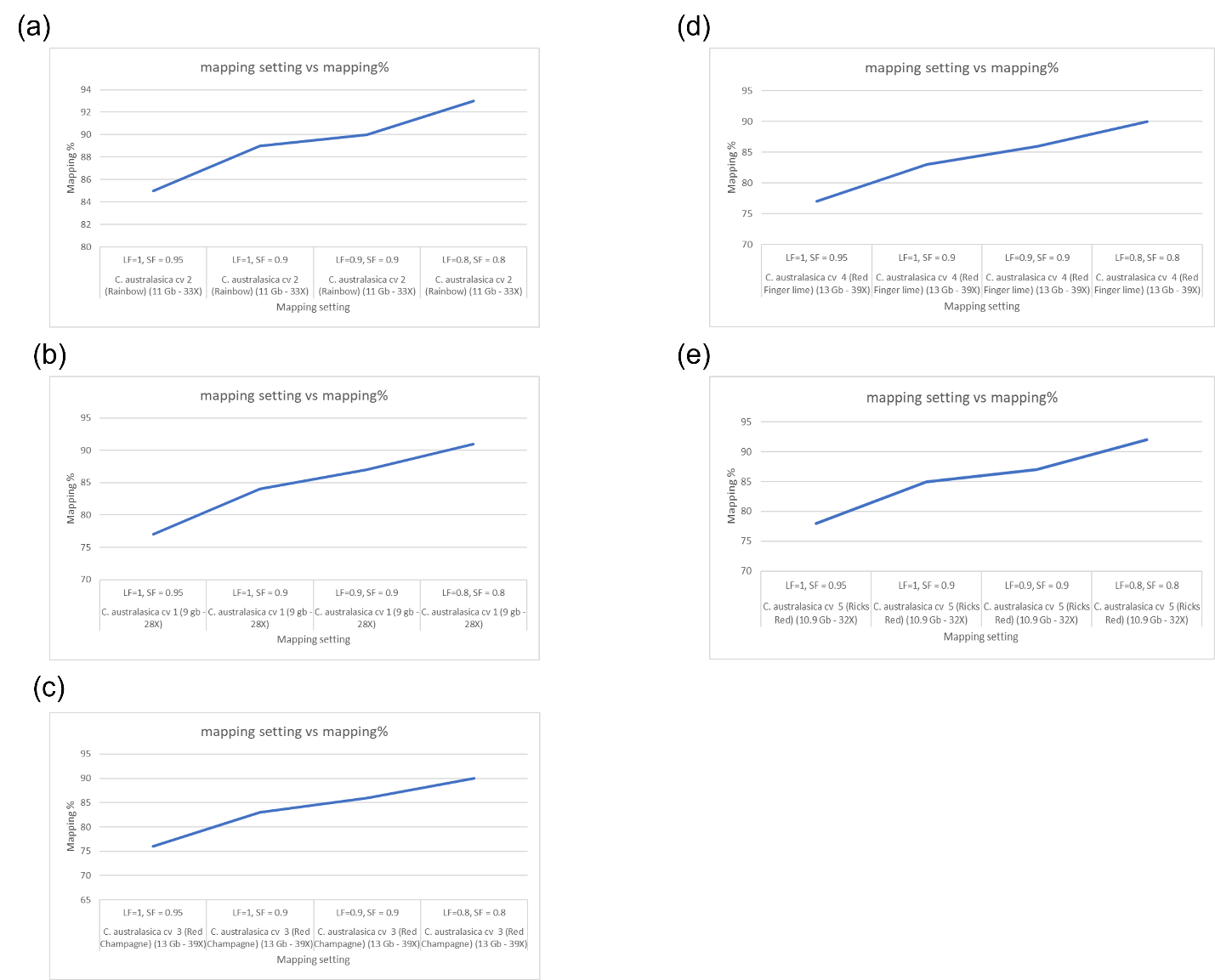


**Figure S2** The mapping of whole genome short reads of five *C. australasica* cultivars to *C. australasica* cv 2 (Rainbow) genome. The reads were mapped using the mapping options of Match score (1), Mismatch cost (2), Linear gap cost [Insertion cost (3), Deletion cost (3)], Auto-detect paired distances – yes, Non-specific match handling – map randomly. Four different mapping stringencies were used; 1. LF=1, SF=0.95, 2. LF=1, SF=0.9, 3. LF=0.9, SF=0.9, 4. LF=0.8, SF=0.8 to map the short reads of (a) *C. australasica* cv 2 (Rainbow) (b) *C. australasica* cv 1 (c) *C. australasica* cv 3 (Red Champagne) (d) *C. australasica* cv 4 (Red Finger lime) (e) *C. australasica* cv 5 (Ricks Red) to Rainbow genome. Read mapping was performed using CLC Genomics Workbench v23,0.4 (Qiagen, USA).


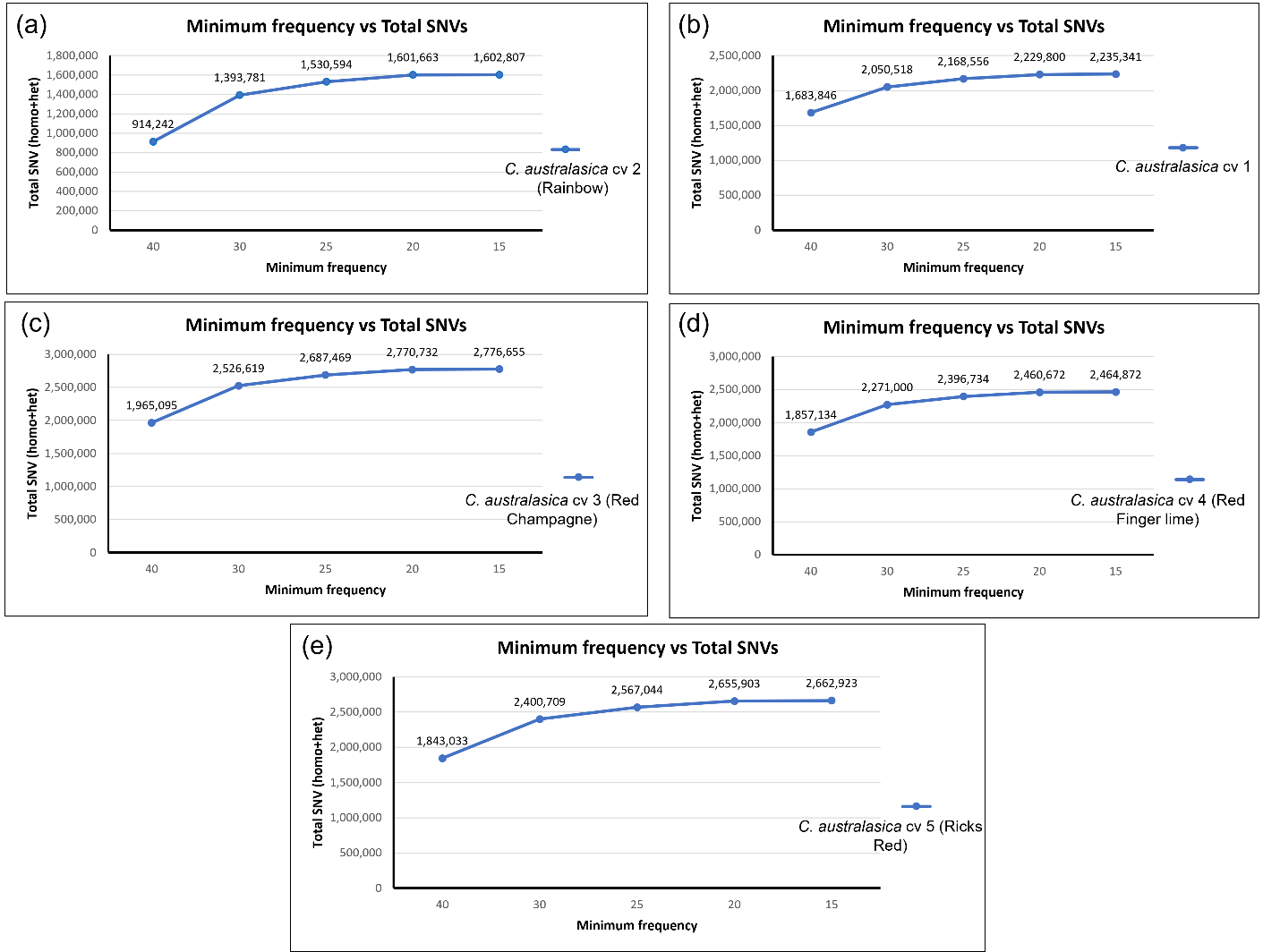


**Figure S3** 100% homozygous and heterozygous SNVs detected at five different minimum frequencies of Fixed ploidy variant detection (FPVD) tool for (a) *C. australasica* cv 2 (Rainbow) (b) *C. australasica* cv 1 (c) *C. australasica* cv 3 (Red Champagne (d) *C. australasica* cv 4 (Red Finger lime) (e) *C. australasica* cv 5 (Ricks Red). FPVD was performed using CLC Genomics Workbench v23,0.4 (Qiagen, USA).


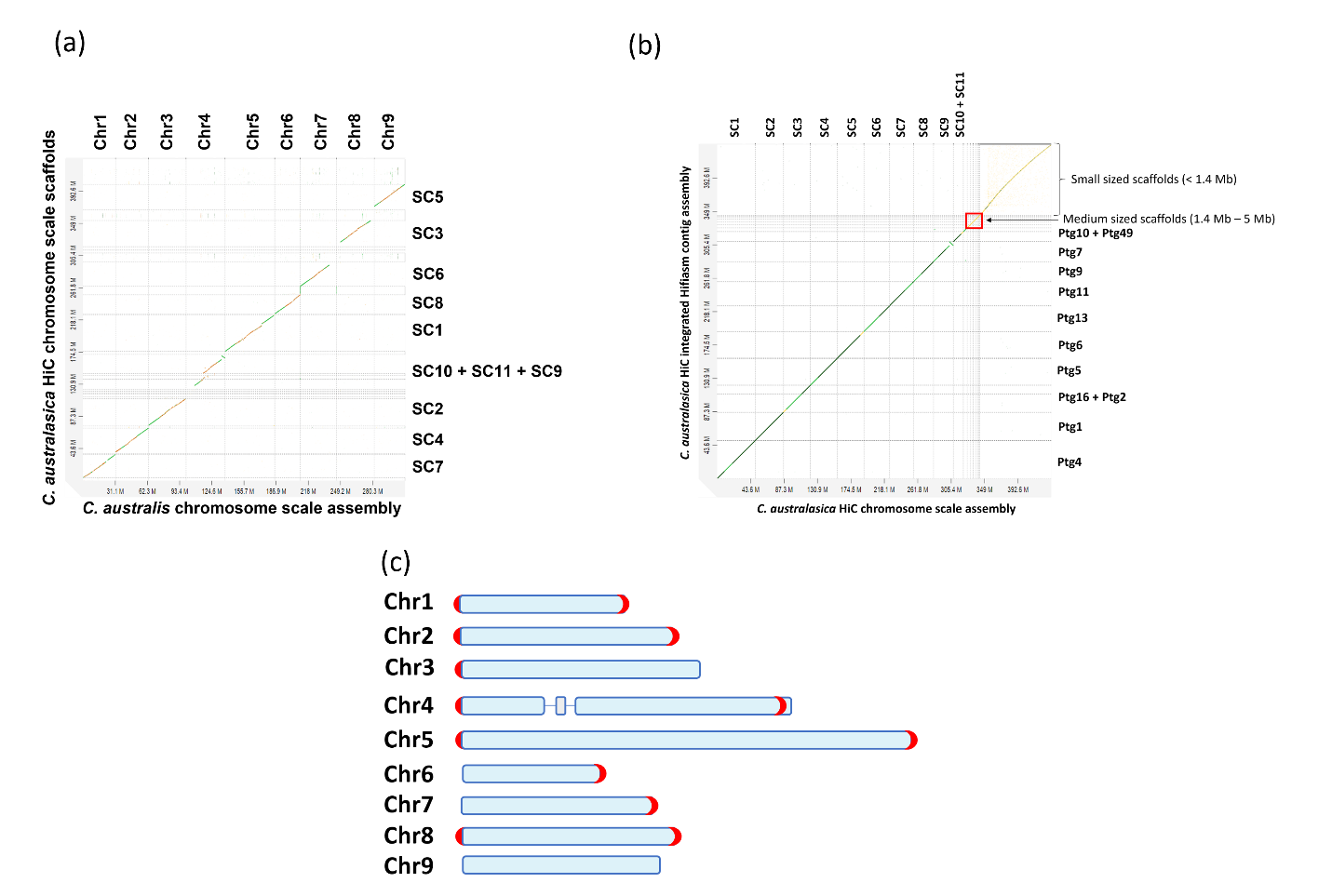


**Figure S4** The alignment of *C. australasica* scaffolds with *C. australis* and the contig composition and characteristics of *C. australasica* pseudochromosomes (a) The assignment of *C. australasica* scaffolds to chromosomes based on the alignment with *C. australis* chromosomes. Three scaffolds (SC10, SC11, SC9) could be assigned to Chr4 based on the alignment with *C. australis* genome (b) The contig composition of each scaffold of *C. australasica.* SC3 (Chr8) was composed of two contigs and all other scaffolds contained single contigs based on Hi-C. (c) The rectangular shapes represent pseudochromosomes, and the lines in Chr4 indicate “Ns” which were used to join nearby scaffolds. The presence of telomeres at the terminal regions of 9 chromosomes of *C. australasica* are shown as red-capped ends*.* Of nine pseudochromosomes, four had telomeres at both terminals, whereas three had telomeres at one terminal. In Chr4, the resulted pseudomolecule had a telomere at one end of SC10 and another telomere at peri terminal region of SC9. Chr9 had no telomeres at either end. The alignments were done using D-Genies (https://dgenies.toulouse.inra.fr/).


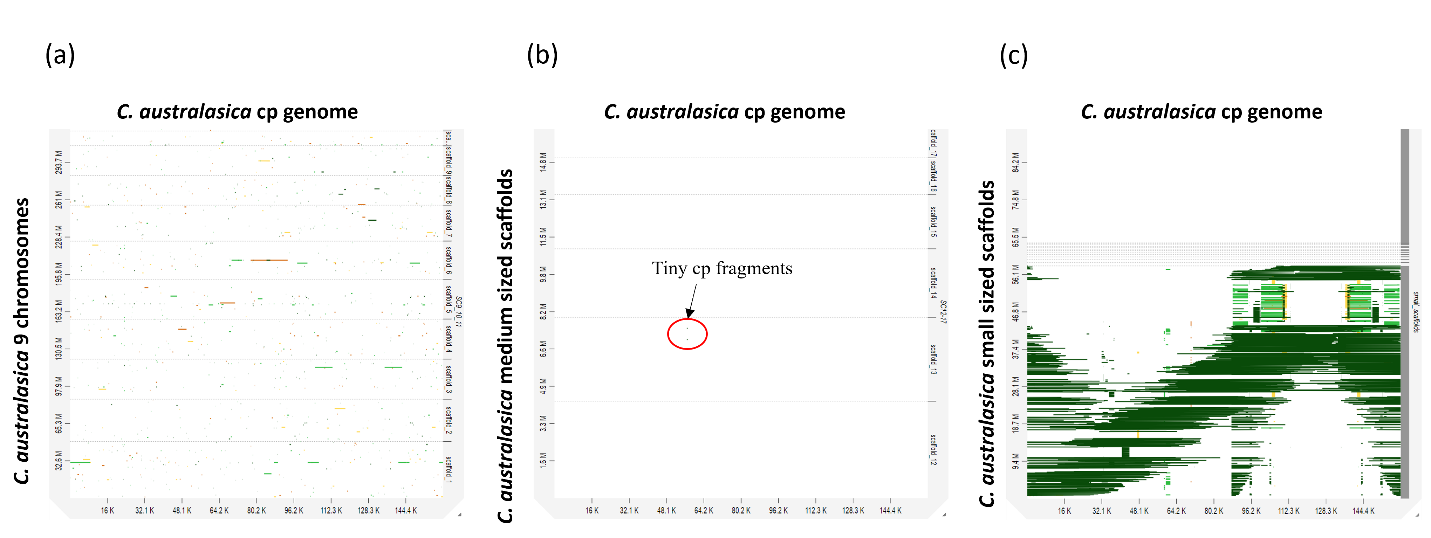


**Figure S5** The alignment of three sets of scaffolds with *C. australasica* chloroplast (cp) genome. (a) The insertion of tiny cp fragments within large chromosome scale pseudomolecules. (b) The insertion of tiny cp fragments within medium sized scaffolds (5 Mb – 1.4 Mb). The alignment of small scaffolds (< 1.4 Mb) with *C. australasica* cp genome indicates that these tiny scaffolds primarily represent the cp genome. The alignments were done using D-Genies (https://dgenies.toulouse.inra.fr/).


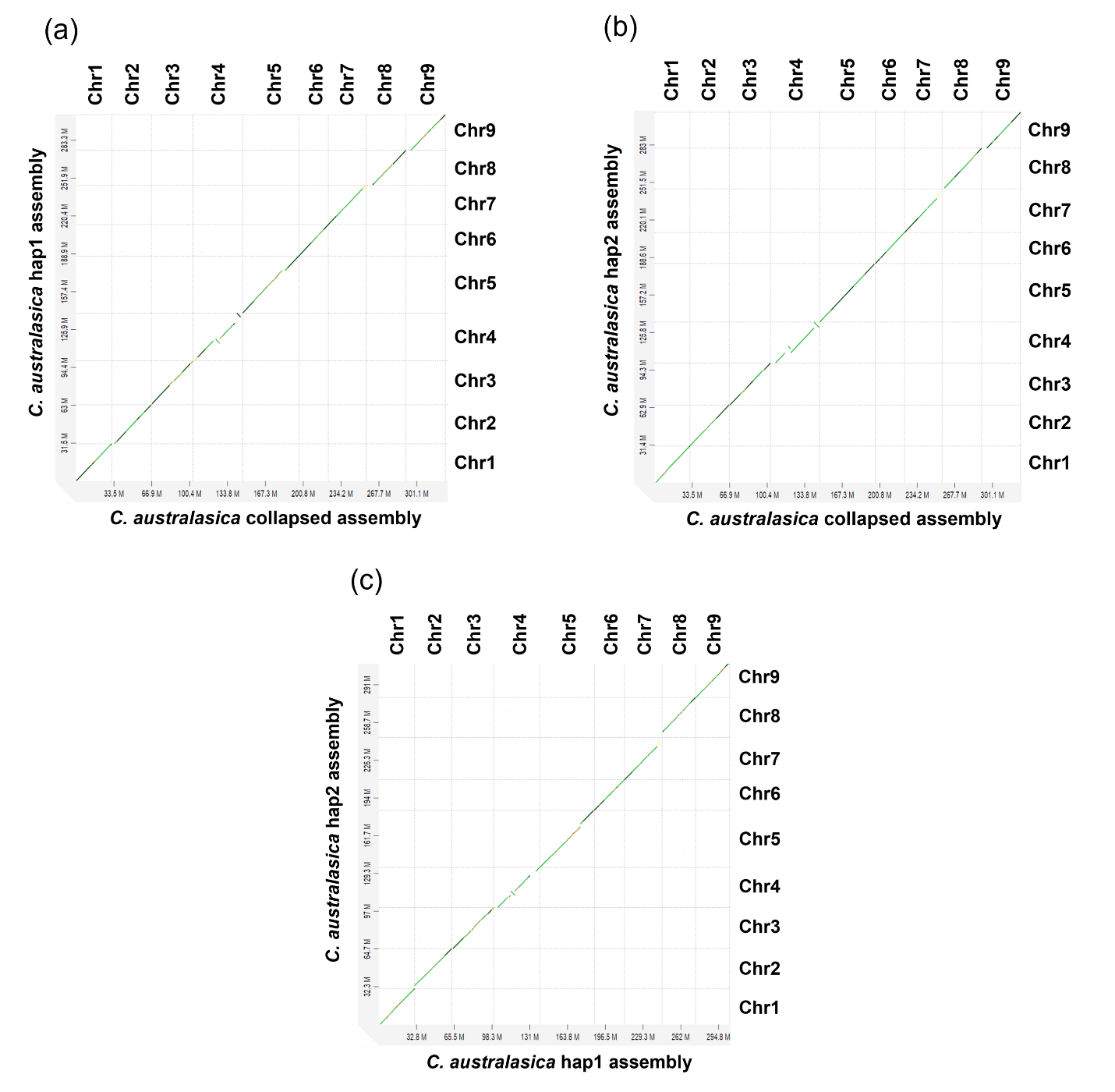


**Figure S6** The alignment of *C. australasica* haplotypes with collapsed assembly (a) The alignment of collapsed assembly vs hap1 assembly (b) The alignment of collapsed assembly vs hap2 assembly (c) The alignment of hap1 assembly vs hap2 assembly. The alignments were done using D-Genies (https://dgenies.toulouse.inra.fr/).


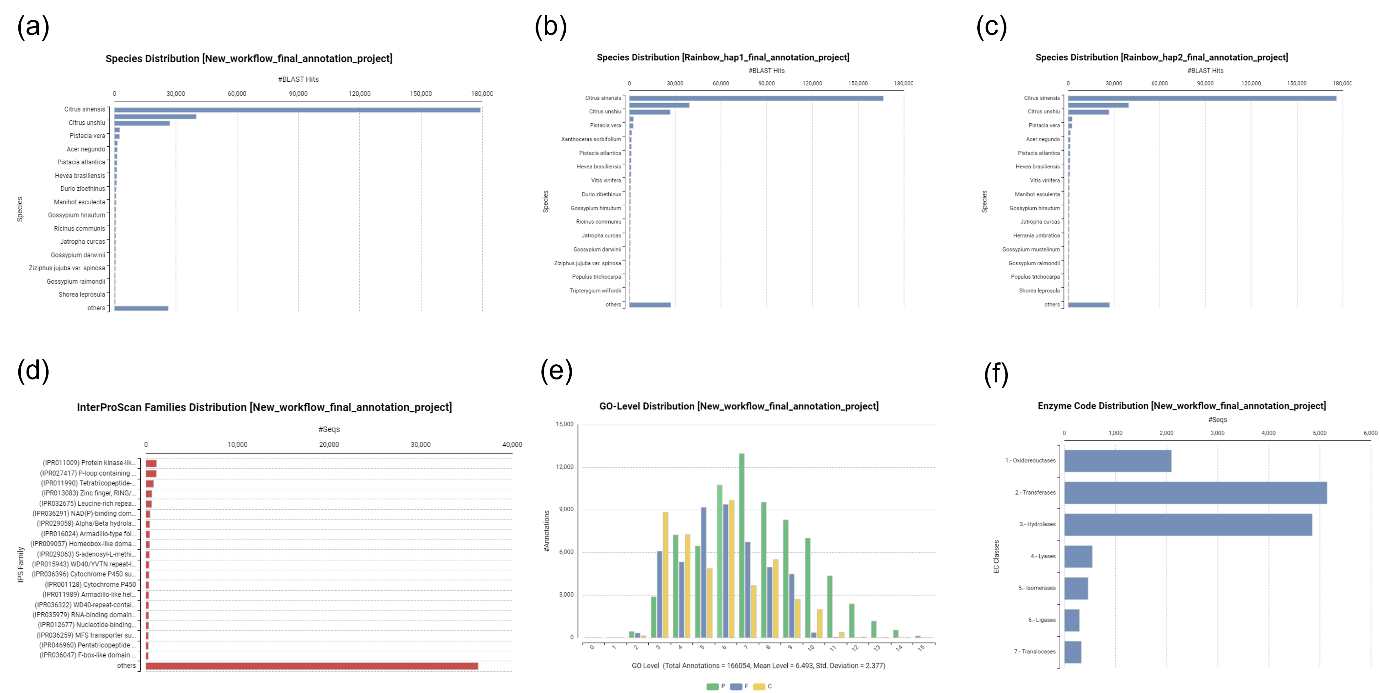


**Figure S7** Functional characterization of CDS sequences in *C. australasica* collapsed, hap1 and hap2 genomes using Omics Box 3.0.30. (a) A list of different species to which most sequences in collapsed genome were aligned during the BLAST step. The maximum number of sequences received BLAST hits from *C. sinensis*, *C. clementina* and *C. unshiu* (b) A list of different species to which most sequences in the hap1 genome were aligned during the BLAST step (c) A list of different species to which most sequences in the hap2 genome were aligned during the BLAST step (d) Bar chart representing the number of sequences in the collapsed genome that belong to a particular IPS family (e) A bar chart which shows all GO terms for all 3 categories (biological process, molecular function and cellular component) for a given GO level in the collapsed genome (f) Distribution of the seven main enzyme classes over all sequences in the collapsed genome.

**
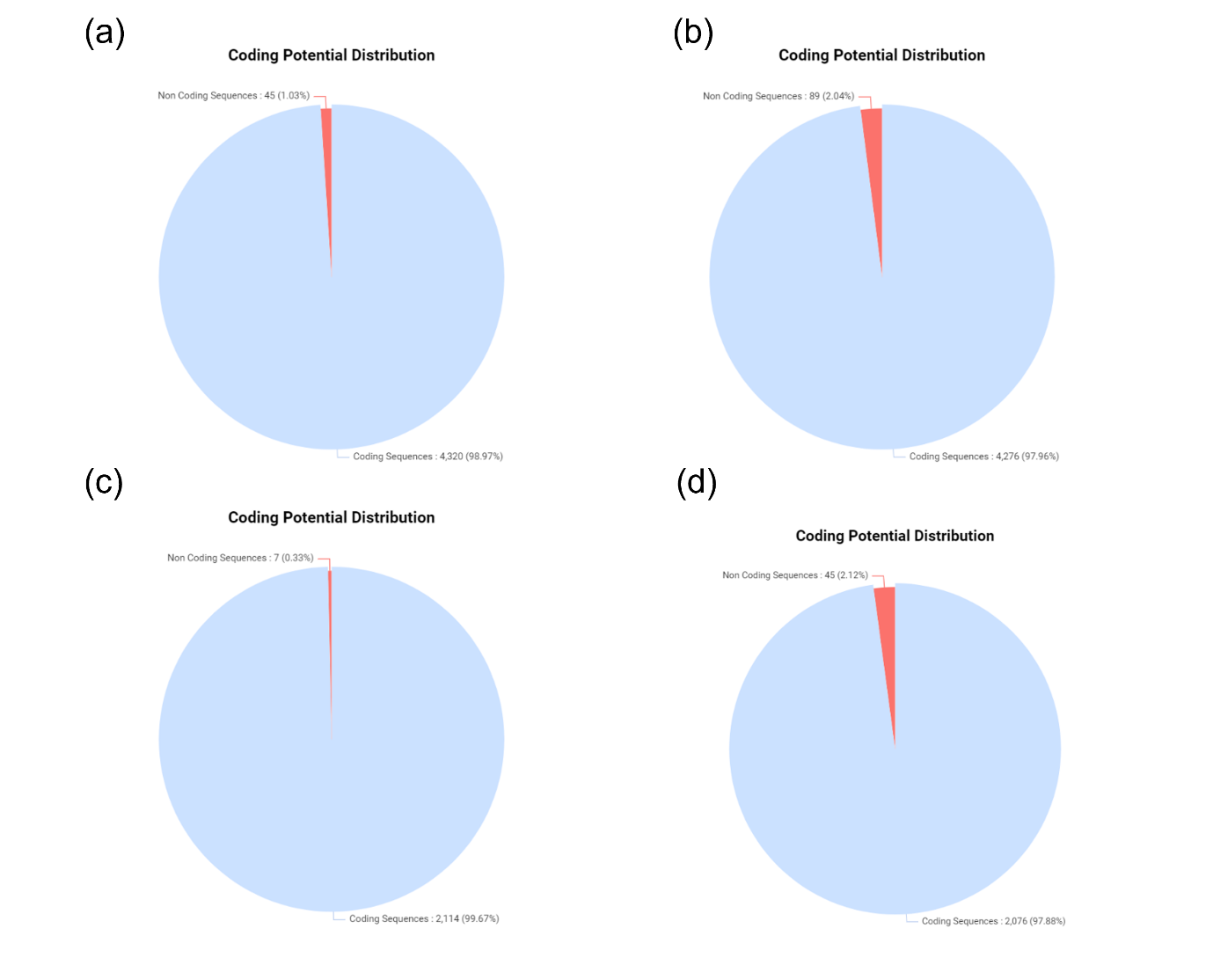
**

**Figure S8** The coding potential of the sequences with no BLAST hits for the two haplotypes **(**a) The coding potential assessment for 4365 hap1 sequences with no BLAST hits. 98.97% of the sequences have coding potential based on models from coding and non-coding sequences of Arabidopsis thaliana (b) 99.07% hap1 sequences have coding potential based on models from coding and non-coding sequences of Citrus. (c) The coding potential assessment for 2121 hap2 sequences with no BLAST hits. 99.67% of the sequences have coding potential based on models from coding and non-coding sequences of Arabidopsis thaliana (d) 97.88% hap2 sequences have coding potential based on models from coding and non-coding sequences of Citrus. Coding potential assessment was done using Omics Box 3.0.30.

**
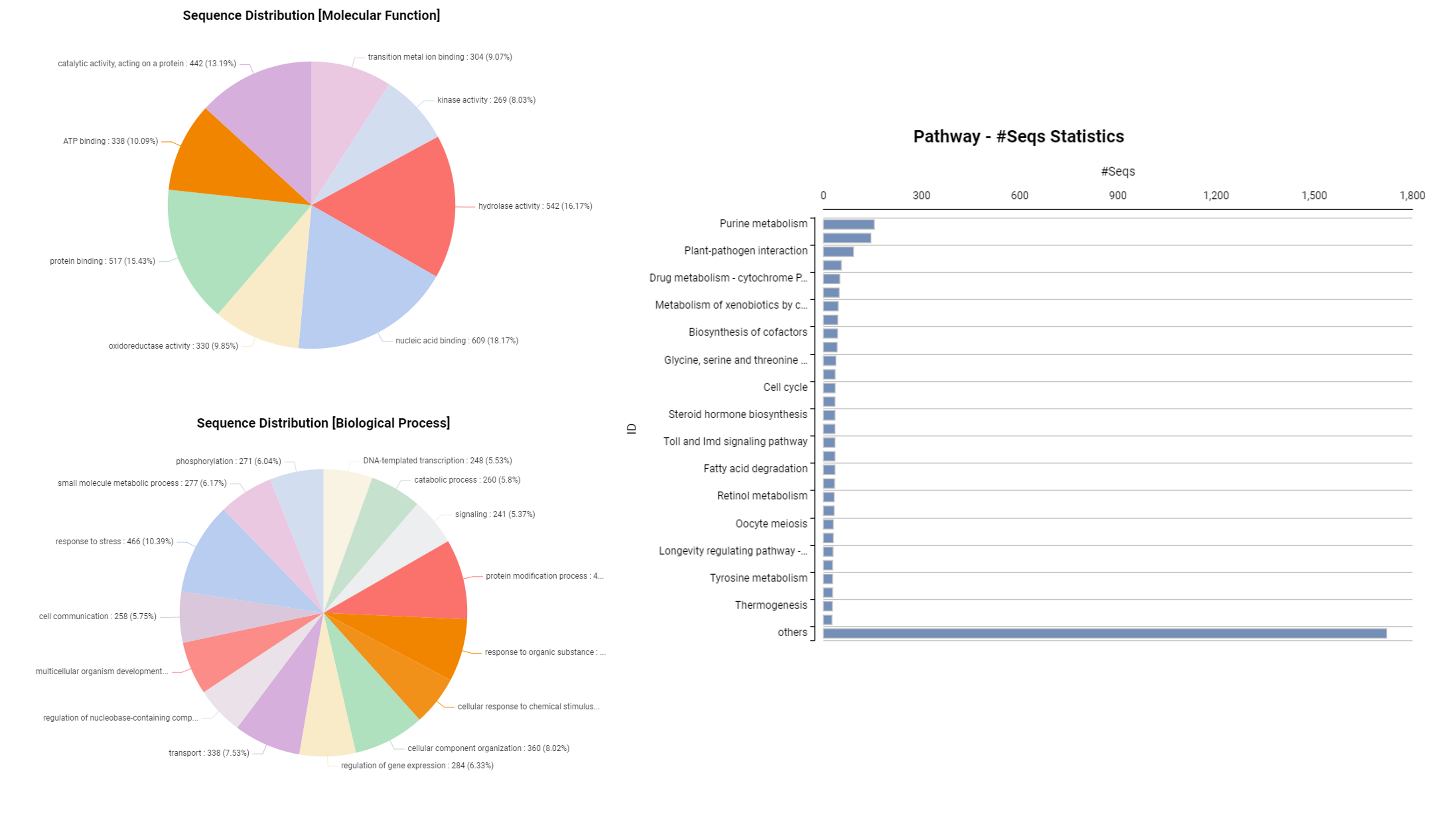
**

**Figure S9** The functions of 12,748 unique protein coding genes in *C. australasica*. (a) The molecular functions (b) The biological processes (c) pathways associated with unique genes. The functions of the unique genes were retrieved by combined graph module in Omics Box 3.0.30.


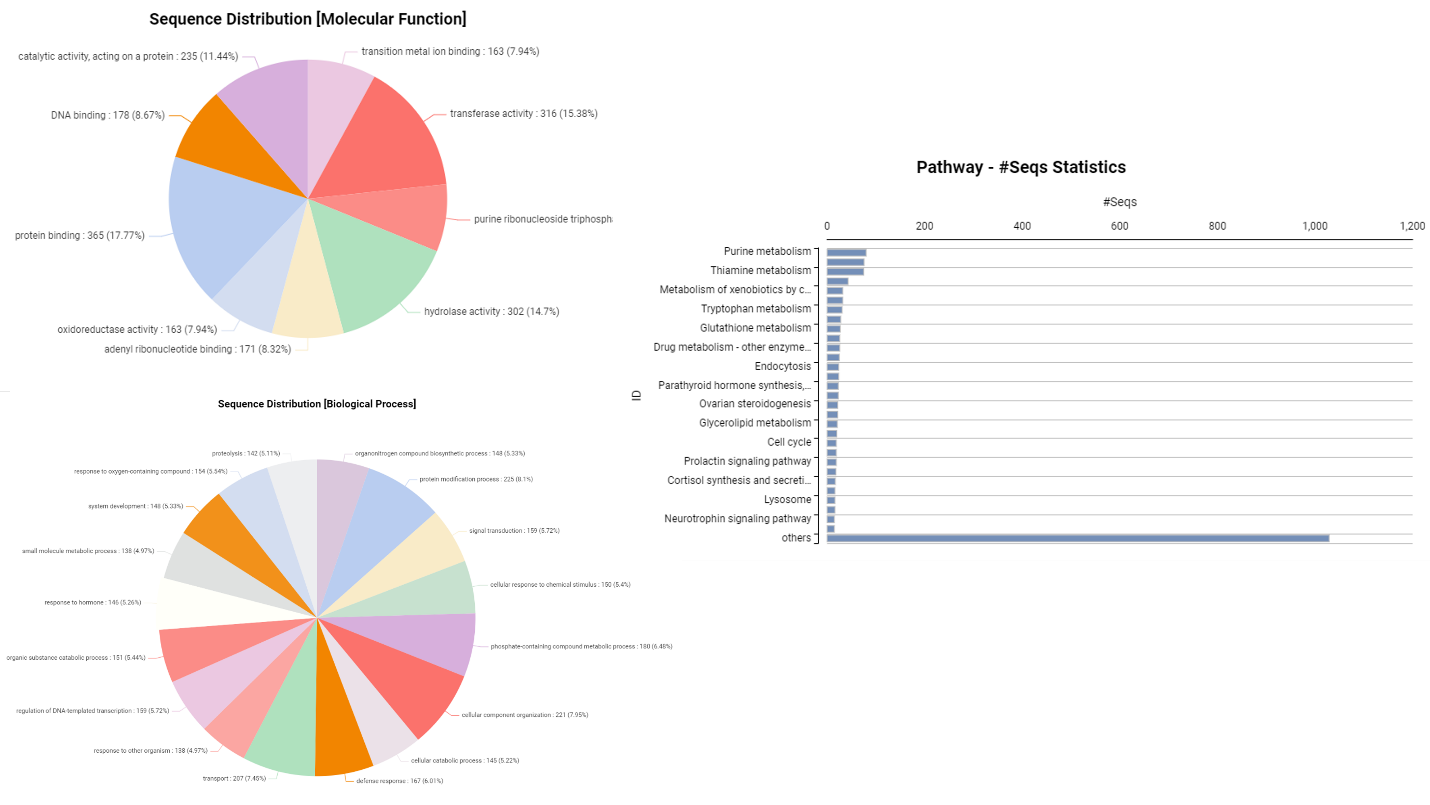


**Figure S10** The functions of 4191 unique protein coding genes in *C. australis*. (a) The molecular functions (b) The biological processes (c) pathways associated with unique genes. The functions of the unique genes were retrieved by combined graph module in Omics Box 3.0.30.


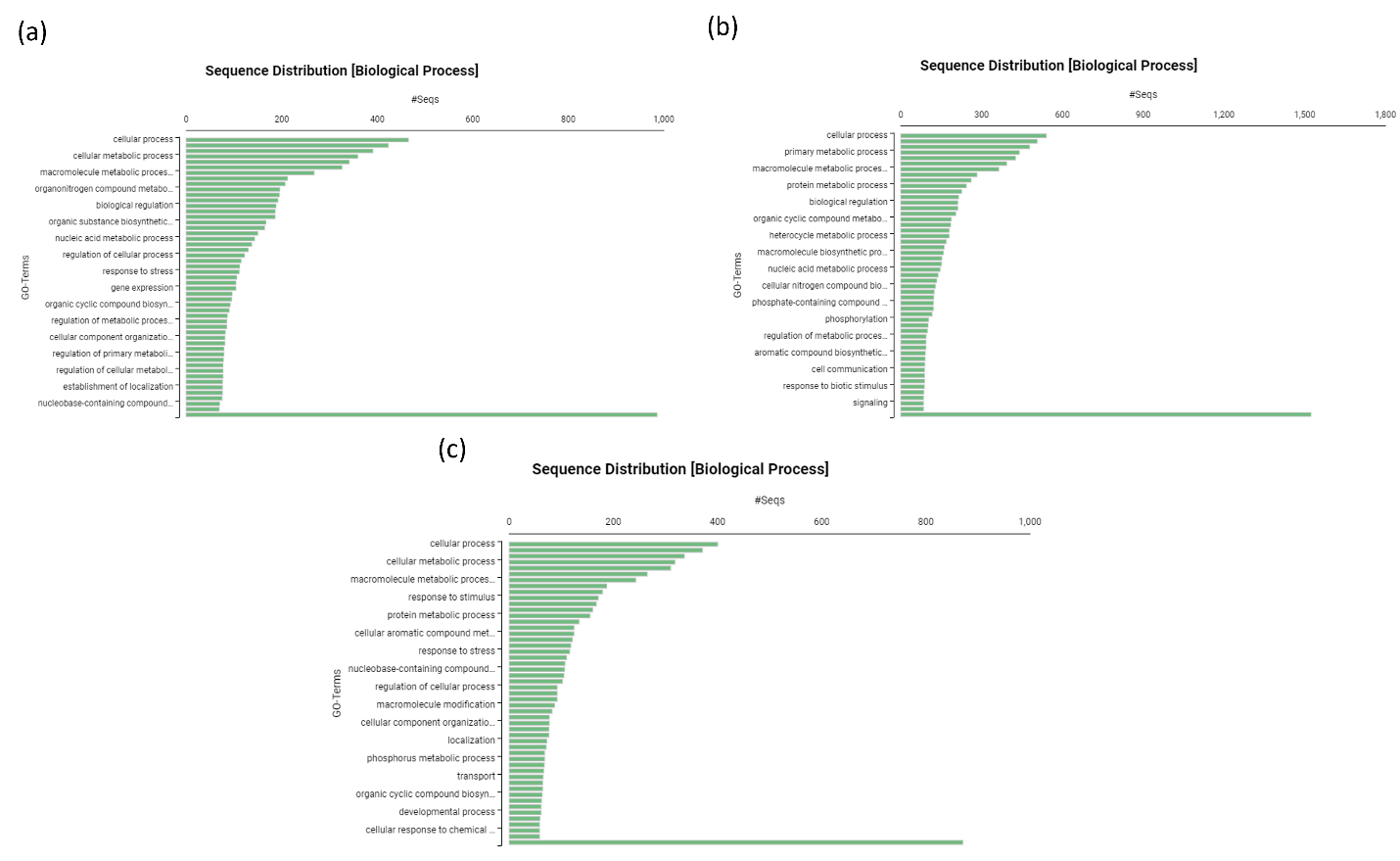


**Figure S11** The functional characterization of *C. australasica* collapsed genome and two sub genomes specific genes. (a) The sequence distribution of collapsed unique genes (b) hap1 unique genes (c) hap2 unique genes for different gene ontology terms related to biological processes. The functions of the unique genes were retrieved by combined graph module in Omics Box 3.0.30.

**
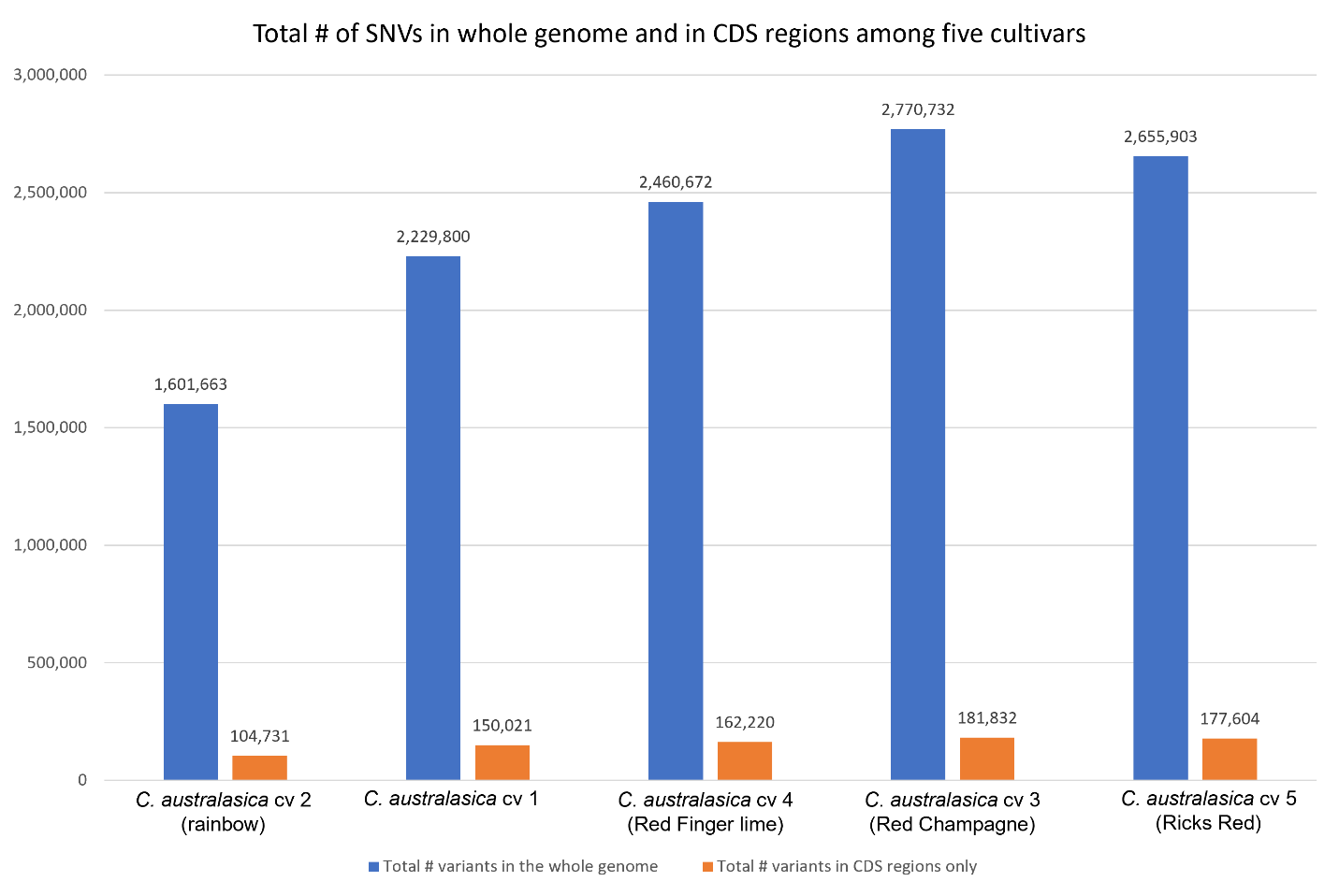
**

**Figure S12** The total number of SNVs (heterozygous and 100% homozygous) in the whole genome and in CDS regions of five *C. australasica* cultivars. The blue indicates the number of SNVs present in the whole genome and orange indicate those present in CDS regions. The highest number of SNVs identified in *C. australasica* cv 3 and the lowest number of SNVs identified in *C. australasica* cv 1 with respect to Rainbow genome. FPVD was performed using CLC Genomics Workbench v23,0.4 (Qiagen, USA).


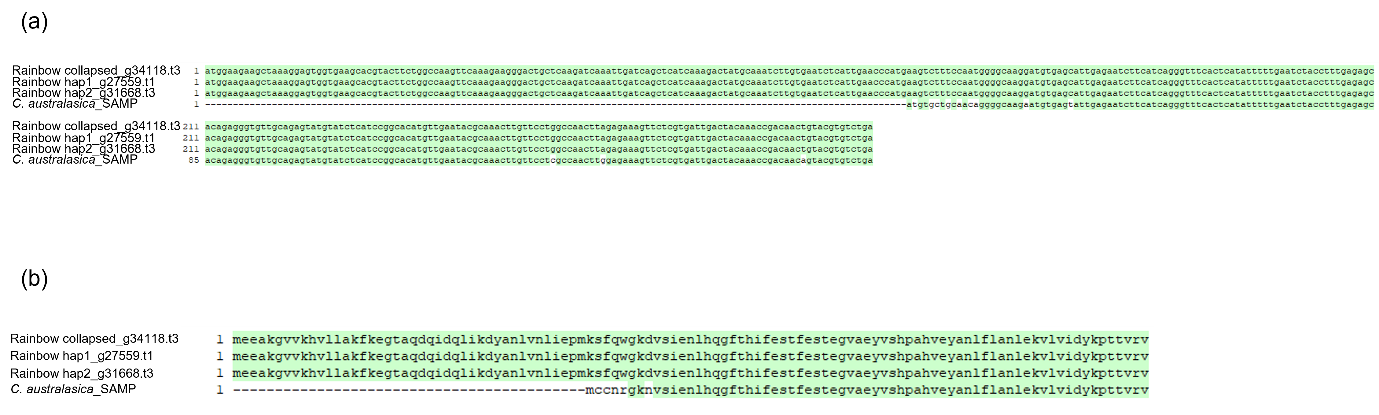


**Figure S13** The alignments of CDS and the corresponding antimicrobial peptides in *C. australasica.*

g34118, g27559, g31668 were identified in three Rainbow assemblies. SAMP, previously reported from *C. australasica*. Alignments between stress-responsive A/B barrel domain-containing proteins encoding genes (a) and corresponding peptides (b). The alignments were performed in Clone Manager Ver. 9.


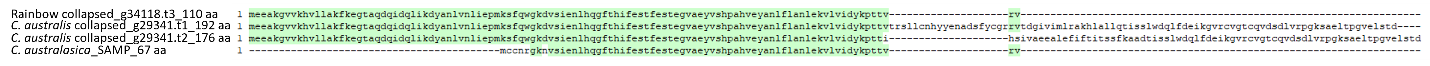


**Figure S14** The alignment of SAMP homologs identified in *C. australasica* and *C. australis.* The two SAMP homologs of *C. australis* had homology with 67 short SAMP sequence of *C. australasica*. The alignments were performed in Clone Manager Ver. 9.


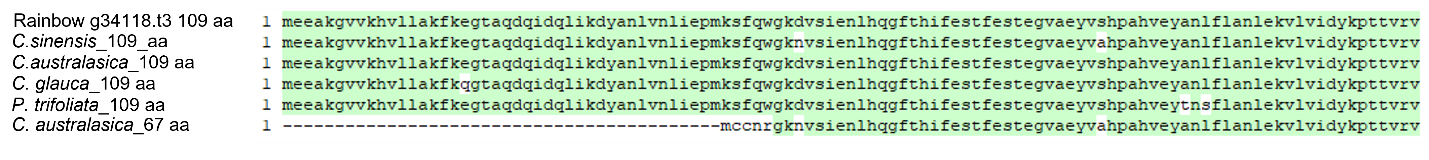


**Figure S15** The alignment between long versions of SAMP sequences in HLB resistant and susceptible citrus species including Rainbow g34118.t3 peptide with the short version of SAMP sequence identified in previous *C. australasica*. The long versions of SAMP sequences of all resistant and susceptible cultivars including *C. australasica* sequence of present study (109 bp) had high homology with each other. They all had high sequence homology with the short version of SAMP sequence of *C. australasica* identified in the previous study. The alignments were performed in Clone Manager Ver. 9.


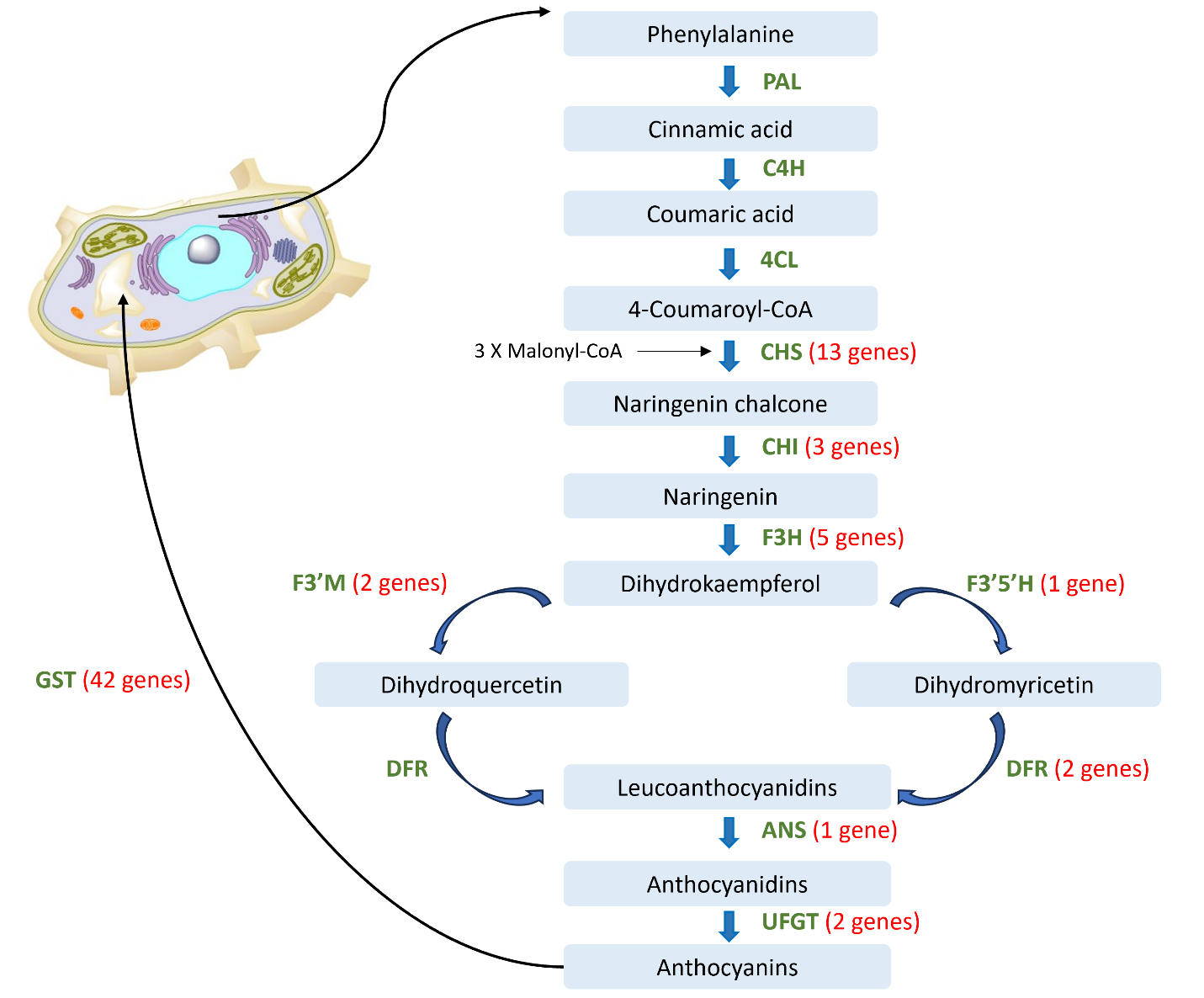


**Figure S16** – Anthocyanin biosynthetic pathway. Anthocyanin biosynthesis initiates with Phenylalanine as the direct precursor which is converted to cinnamic acid by phenylalanine ammonia lyase (PAL). Subsequently, cinnamic acid is converted to coumaric acid by 4-hydroxylase (C4H) which is then converted to the respective CoA ester by 4-coumarate: CoA ligase (4CL). Chalcone synthase (CHS) then condenses 4-Coumaroyl-CoA and Malonyl-CoA to generate naringenin chalcone which is isomerized by chalcone isomerase (CHI) to flavanone naringenin by chalcone isomerase (CHI). Naringenin is then converted to dihydrokaempferol by flavanone 3′-hydroxylase (F3H) which then synthesizes dihydroquercetin and dihydromyricetin by Flavonoid 3'-monooxygenase (F3’M) and flavonoid 3′,5′-hydroxylase (F3’5’H) respectively. The corresponding colourless leucoanthocyanidins are then produced by Dihydroflavonol 4-reductase (DFR), which are then converted to coloured anthocyanidins (ANS). The stability and the hydrophilicity of anthocyanidins are increased by UDP-glucoseflavonoid glucosyl transferase (UFGT). In the last step, a specific glutathione transferase (GST) mediates the transfer of anthocyanins to vacuole. The pathway analysis was performed using KEGG in OmicsBox 3.0.30. The plant cell image was taken from ChemDraw 20.1.1.

**Method S1**

Scaffolding was performed with Hi-C data using three scaffolding tools using two different pipelines.

In the first option, we used Bowtie2 aligner to align the Hi-C reads against the indexed contig level genome and the genome was scaffolded using SALSA scaffolder. The whole assembly generated from it had 31.4 Mb N50. Only three scaffolds had telomeres at two terminals whereas, eleven scaffolds had telomeres at one terminal region. Scaffolds that could be assigned into chromosome level based on the alignments with *C. australis* are only shown in Supplementary Table2. The top 12 scaffolds are required to cover the total number of BUSCOs of the assembly (Supplementary Table2a). In the 2nd option, we aligned the Hi-C reads to the genome with Bowtie2 aligner followed by scaffolding using YaHS tool. The top two scaffolds are relatively large (SC1 - 64 Mb and SC2 - 62 Mb respectively) due to the merging of two large contigs together which is not comparable with our reference genome (*C. australis*). In this system, different portions of the same contig have been considered as different scaffolds (eg1: ptg 11 – SC6, SC10, eg2: ptg 7 is a part of SC7 and SC11) and some scaffolds were corresponded to more than one chromosome which is not reliable (SC1 correspond to Chr2 and Chr7) (Supplementary Table2b).

In the 3rd option, we used Chromap aligner with SALSA. With this system, there were four scaffolds with telomeres at both terminals, eight scaffolds with telomeres at one end and one scaffold with an interior telomere. There were four scaffolds corresponding to Chr4 based on *C. australis* genome, with two scaffolds (SC10, SC11) with telomeres at one terminal (Supplementary Table2c). In the 4th option, Hi-C alignment was done using Chromap and the scaffolding was performed using YaHS tool. There were four scaffolds with telomeres at both ends including one scaffold with an interior telomere (Supplementary Table2d).

In the 5th option, we used BWA aligner to align the Hi-C reads through Arima mapping pipeline where it only retains the 5’ side of the chimeric reads and removes PCR duplicates. The alignment.bam file was then subjected to SALSA for scaffolding. This pipeline generated four scaffolds with telomeres at both terminals and nine scaffolds with telomeres at one terminal. Three scaffolds (SC10, SC11, SC9) could be assigned to Chr4 and the resulted pseudomolecule had a telomere at one end of SC10 and another telomere at peri terminal region of SC9. A total of 11 scaffolds could cover 99% complete BUSCOs. There were some scaffolds (SC10, SC11, SC8 and SC20) with 5.8S and 28S rRNA gene repeats at terminal regions and some scaffolds with high copy number tandem arrays of satellite DNA repeats at their terminal regions. The presence of rRNA gene repeats and satellite repeats at the terminal regions might be the main reason for not being able to assemble certain pseudomolecules as complete chromosomes. The assembly contiguity is 33.5 Mb (N50). The results of this pipeline are the best among all methods we checked (Supplementary Table2e).

Then the same pipeline was performed using YaHS as the scaffolder. The assembly N50 is slightly better than SALSA (34.3 Mb), however, there is an internal telomere in one of the scaffolds. Three scaffolds have telomeres at both ends, one of which (SC3) could be assigned to Chr4 together with another scaffold, therefore, having one interior telomere if the two scaffolds jointly represent the Chr4. There were seven scaffolds with telomeres at one terminal (Supplementary Table2f). The same pipeline was next performed with pin_hic scaffolder. The results were similar to those from SALSA. Both SALSA and pin_hic tools have selected the same contigs or merged two contigs corresponding to each chromosome although the scaffold IDs were different. Therefore, either BWA+Arima mapping+SALSA or BWA+Arima mapping+pin_hic could be selected as the final assemblies. We proceeded with BWA+Arima mapping+SALSA which is mentioned as the scaffold assembly in the main manuscript.

The scaffolds were renamed and oriented based on *C. australis* genome. Four scaffolds had telomeres at both ends and they were assigned as Chr1, Chr2, Chr5, and Chr8 (Supplementary Table 2e). Chr8 was composed of two contigs (ptg16 and ptg2) which were joined together with 500Ns in between them by SALSA. The dotplot alignment of finger lime scaffolds and *C. australis* chromosomes identified three scaffolds of finger lime that belong to Chr4, therefore 500Ns were manually added in between the three scaffolds to form Chr4 of finger lime. Chr4 had one telomeric repeat at one end another telomeric repeat at the other peri-telomeric region. Chr3 had a telomere which was not as a continuous sequence, however there were few telomeric repeats were present intermittently at the end of the chromosome. Scaffold 8 had telomere at one end, and 5.8S and 28S ribosomal repeats at the other end. There were four small scaffolds (SC13, SC14, SC18 and SC20) which were ≤ 3.7 Mb and with telomeres at one end, and one of them (SC20) had 5.8S and 28S ribosomal repeats at the other end. Hifi reads have been unable to assemble some complete chromosomes due to the presence of large clusters of rRNA genes repeats or large tandem arrays of satellite repeats. All the small scaffolds with telomere at one end were kept as separate sequences and were annotated along with nine chromosomes as they were also could be parts of nuclear genome sequence, however, couldn’t assigned into an exact chromosomal location with the available linkage data.
